# Rapid Discrimination Among Putative Mechanistic Models of Biochemical Systems

**DOI:** 10.1038/srep32375

**Published:** 2016-08-31

**Authors:** Jason G. Lomnitz, Michael A. Savageau

**Affiliations:** 1 Department of Biomedical Engineering, University of California, Davis, CA 95616, USA; 2 Department of Microbiology & Molecular Genetics, University of California, Davis, CA 95616 USA

## Abstract

An overarching goal in molecular biology is to gain an understanding of the mechanistic basis underlying biochemical systems. Success is critical if we are to predict effectively the outcome of drug treatments and the development of abnormal phenotypes. However, data from most experimental studies is typically noisy and sparse. This allows multiple potential mechanisms to account for experimental observations, and often devising experiments to test each is not feasible. Here, we introduce a novel strategy that discriminates among putative models based on their repertoire of qualitatively distinct phenotypes, without relying on knowledge of specific values for rate constants and binding constants. As an illustration, we apply this strategy to two synthetic gene circuits exhibiting anomalous behaviors. Our results show that the conventional models, based on their well-characterized components, cannot account for the experimental observations. We examine a total of 40 alternative hypotheses and show that only 5 have the potential to reproduce the experimental data, and one can do so with biologically relevant parameter values.

An important subclass of biological systems is concerned with making logical decisions from a number of input signals. Such systems play important roles in diverse cellular processes that span multiple levels of biological organization. For example, the underlying strategy for much current research in stem cell biology is an attempt to identify the relevant input signals and the mechanisms by which they are processed to produce a reliable outcome, often one of two different cell fates at any given stage^1^. The process of lineage stratification, as in hematopoiesis, often follows a series of logical decision-making steps involving different morphogen signals^2^. At the transcriptional level, genes are regulated by complex mechanisms that involve many different factors to ensure that transcription only occurs under carefully controlled conditions. The classic example is catabolite repression of the *lac* operon, where the *cis*-regulatory control element achieves maximal promoter activity only when both allolactose and cAMP concentrations are high, effectively operating as an AND logic gate^3^.

The task of understanding the mechanistic basis underlying complex biological phenomena is challenging, and often involves different strategies that include designing specific experiments, analyzing large amounts of data and developing mathematical models. One promising strategy to gain insight into the operation of natural systems involves the use of small synthetic constructs that capture the essential interactions^4^,^5^. For example, synthetic biology has been used extensively to understand genetic regulatory circuits that implement a binary logic-gate^6^,^7^,^8^,^9^. However, as systems become complex, conceptual models and intuitive approaches to uncover their implications are no longer adequate, and it becomes necessary to formulate mathematical models and apply more rigorous methods. This is most apparent in the context of synthetic constructs that exhibit unexpected results and yet they consist of small numbers of components that are typically among the best studied in all of biology^7^,^9^. The problem is compounded if the available data are sparse, as is most often the case. As a result, there are potentially many models that might account for the observations, and our ability to discriminate among the models is limited.

Testing numerous putative hypotheses experimentally can be difficult and time consuming. Mathematical modeling provides an alternative approach for rapid elimination of hypotheses with serious but often subtle inconsistencies. Conventional modeling strategies involve fitting data in order to estimate parameter values and validating mechanistic models against a set of independent measurements [e.g. ref. 10]. However, the parameters of complex kinetic models are difficult to estimate, and this approach cannot determine if a behavior is possible in a manner that is independent of parameter values.

Here, we present a novel strategy aimed at overcoming this challenge; it consists of three phases: (1) an innovative model-independent phase that rigorously characterizes experimental data, (2) a model-dependent phase that automatically identifies the repertoire of model phenotypes, and (3) a model-discrimination phase that systematically compares the results of the previous phases. The methods are first presented in detail using a simple well-characterized system, the *lac* operon of *Escherichia coli,* to facilitate the introduction.

We then illustrate this strategy with an application to published examples of synthetic gene constructs that exhibit unexpected results^7^, and demonstrate that the conventional models are unable to exhibit specific experimental observations, regardless of parameter values. We propose a series of hypotheses with the potential to resolve the discrepancies between model phenotypes, as defined rigorously within the System Design Space method^11^,^12^, and experimental data. Our analysis identifies 5 out of 40 hypotheses that can account for the qualitative nature of the experimental observations. Thus, given experimental results consisting of discrete observations and a set of mechanistic models based on biochemical kinetics, our strategy determines whether the models are consistent or inconsistent with the experimental data.

## Three-Phase Strategy for Model Discrimination

**Figure 1:**
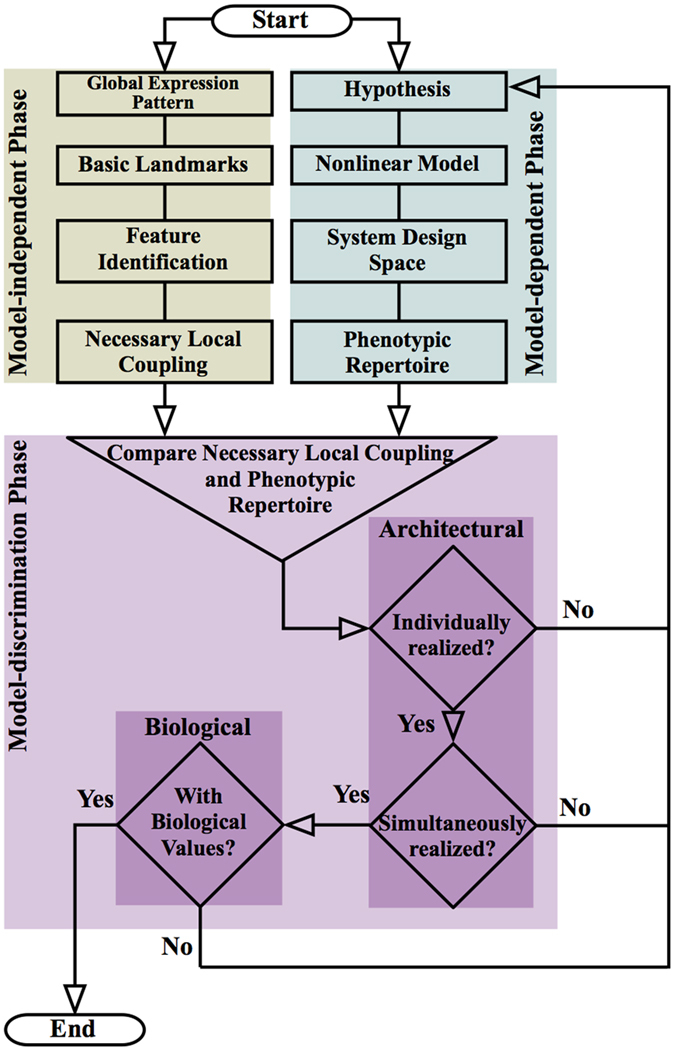
A flowchart illustrating the major steps in the strategy for model discrimination. The three phases of the method are highlighted by different colors. Discrimination at the level of model architecture is determined without parameter values. Discrimination at the biological level is determined in the same way as at the architectural level but with the imposition of bounds on the values for the parameters such that they are biologically relevant.

The process of model discrimination requires comparing model behavior against experimental data. Our strategy, outlined in Fig. 1, involves three phases: analyzing landscapes that realize discrete experimental data to identify their essential landmarks, analyzing quantitative models to identify their repertoire of qualitatively distinct phenotypes, and reconciling landmarks and phenotypes to discriminate among alternative models (hypotheses). The first is a model-independent phase that is concerned only with characterizing discrete experimental observations. The second is a model-dependent phase that is concerned only with model architecture and parameter values. We have previously defined the architecture of a model as the collection of attributes that remain fixed independent of specific values for the parameters that characterize a particular instance^12^. Important architectural features include (a) the network topology of interactions, (b) the signs of the interactions, and (c) the number of binding sites involved in the interactions. The third is a model-discrimination Phase in which a specific model architecture serves as the basis for our mathematical representation of a hypothesis concerning the experimental data. We test hypotheses at two levels: (1) the architectural level, defined purely by the model’s architecture, and (2) the biological level, defined by parameter values restricted to a range about their experimentally measured or estimated value.

In the following subsections we will describe each phase of this three-part strategy using a simple example involving AND binary logic to illustrate the more general abstract concepts.

### Model-Independent Phase

It is a fundamental challenge to determine whether or not a model can reproduce an experimental result^13^,^14^,^15^,^16^. The first step involves identifying the characteristics of the observed data and describing the data in a manner that is rigorous and without ambiguities. The aim of the model-independent phase is to provide such a description, regardless of the mechanism that may be responsible for producing it.

To achieve this, we identify continuous underlying functions that can generate the observed data and identify key landmarks within these functions that may be used for comparison with mathematical models. However, there is a significant challenge that must be overcome: the number of continuous functions capable of generating the observed data can be large to infinite. Our solution is to identify a reduced set of continuous functions, which are analogous to a basis set, that are capable of generating all other functions that can account for the observed data. To make this approach clearer, we introduce the following concepts that are defined below: (1) *landscape*, (2) *feature*, (3) *local coupling,* (4) *landmark,* and (5) *basic landmark*.

**Figure 2:**
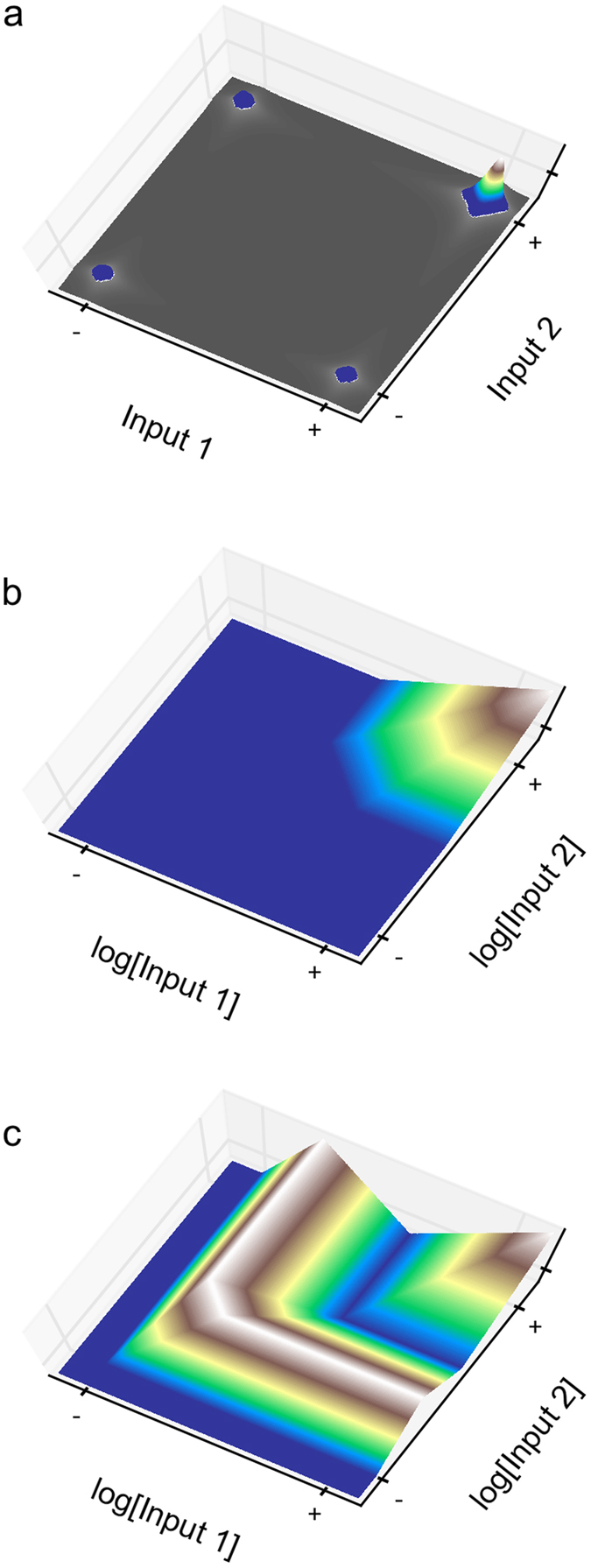
Discrete experimental data representing an AND logic function and two examples with different landscapes that realize the same experimental data. The x- and y-axes represent the concentration of the first and second input variables in logarithmic coordinates, respectively. The z-axis represents the state of the output variable in logarithmic coordinates relative to an arbitrary threshold. Output values represented in brown or white colors correspond to values above the threshold, while all other colors are below the threshold. (**a**) Discrete experimental data representing an AND logic function at discrete input values, where ‘−’ represents a low input value and ‘+’ represents a high input value; gray color corresponds to unknown output values. (**b,c**) two landscapes with different landmarks representing continuous mappings that realize the AND logic function, the ‘−’ and the ‘+’ input values correspond to those in panel (**a**). (**b**) Landscape exhibiting the following features: a peak value at the +/+ state having a monotonic decrease in output with each input separately and with the two inputs simultaneously until the three low states are achieved. (**c**) Landscape exhibiting the following features: a peak value at the +/+ state having a monotonic decrease in output with each input separately until a low valley is reached. On the other side of the valley, the output increases monotonically with each input separately and with the two inputs simultaneously until a ridge is reached at intermediate values of the output. Beyond the ridge, the output decreases monotonically toward each of the three low states.

To facilitate introduction of the more abstract concepts, we use an AND binary logic gate as a concrete example. The AND gate is a function of two input signals and one output signal; when both input signals are high (+/+) the output signal is ‘ON’, and for all remaining combinations of the input signals (+/−, −/+ and −/−) the output signal is ‘OFF’. An example of an AND logic function is shown in Fig. 2a. The AND gate can be clearly defined as having a higher value for the output signal when both input signals are high compared to the remaining combinations of the input signals.

#### Landscape

A *landscape* is defined as the discretized global topography in a multi-dimensional space relating outputs to inputs in a steady- or quasi-steady-state experiment; this definition is independent of any assumed mechanism, and thus is independent of any hypothesis. The landscapes that realize an AND gate with one output and two inputs are defined by having specific output states at four discrete points within a two-dimensional space (Fig. 2).

#### Feature

A *feature* is defined as a local surface or facet within a landscape, again without reference to any assumed mechanism. Features are characterized not only by their location within a landscape but also, equally important, by their gradients relating changes in outputs to changes in the inputs. Three features are evident in the landscape of Fig. 2b, whereas there are many present in the more complex landscape of Fig. 2c.

In addition, we impose a convexity requirement: a feature must be valid within a convex polytope in the logarithmic space of the input variables. This requirement is imposed to facilitate the comparison between the model-independent and the model-dependent phases, as will be shown in the **Model-Discrimination Phase**. We can also impose this restriction on non-convex subspaces by simply decomposing the subspace into a series of convex subspaces.

#### Local coupling

A *local coupling* is defined as one dimension of the multi-dimensional gradient within a feature. This is simply a descriptive aspect of the feature and makes no assumptions about underlying mechanism. The changes observed at the landscape level typically occur through continuous transitions involving distinct local couplings within its features. In the case of an AND gate, we are interested in the change of output as a function of the two inputs. An input and output are directly coupled if an increase (decrease) in the input signal results in an increase (decrease) in the output. An input and output are uncoupled if an increase (decrease) in the input signal produces no change in the output. Finally, an input and output are inversely coupled if an increase (decrease) in the input signal results in a decrease (increase) in the output.

#### Landmark

A *landmark* is defined as a contiguous assemblage of multiple features found within a landscape. Examples of different landmarks can be found in the AND landscapes of Fig. 2. Figure 2b exhibits a single landmark (a peak with alluvial fan) consisting of three features. Figure 2c is an example of a more complex landscape with several landmarks (a peak, a valley, and a ridge with alluvial fans), each with a number of features as described in the legend. If there were only a single landmark with a single feature that could realize the discrete data of an AND gate, then discriminating among hypotheses would be relatively easy. However, there are multiple landmarks that can realize the discrete data of an AND gate; in fact, the number is potentially infinite.

How can we positively discriminate among hypotheses if there are an infinite number of alternative landmarks that can produce a particular experimental result? A solution to this problem involves identifying a subset of simplified landmarks that can serve as a basis for constructing more complex landmarks. By analogy, a Fourier series involving combinations of sine and cosine functions with different phases and amplitudes can represent any periodic function; a linear combination of basis vectors with different magnitudes can represent any vector in a given coordinate system. To represent the infinite set of landmarks in terms of a finite set of basic landmarks, we apply similar principles.

#### Basic landmark

We define a *basic landmark* as a landmark that cannot be decomposed further without losing its ability to realize the discrete experimental data. To identify basic landmarks, we decompose the set of complex landmarks into their constitutive features; then, we ask the following question: can a feature be removed such that the remaining features can be reorganized to produce a set of simpler landmarks that still reproduce the experimental observations? If the answer to this question is yes for any of the features that are removed, then the original landmark having such a feature is a non-basic landmark. Alternatively, if the answer is no for all the features of a given landmark, then it has only essential features and is irreducible; hence, it is a basic landmark.

**Figure 3:**
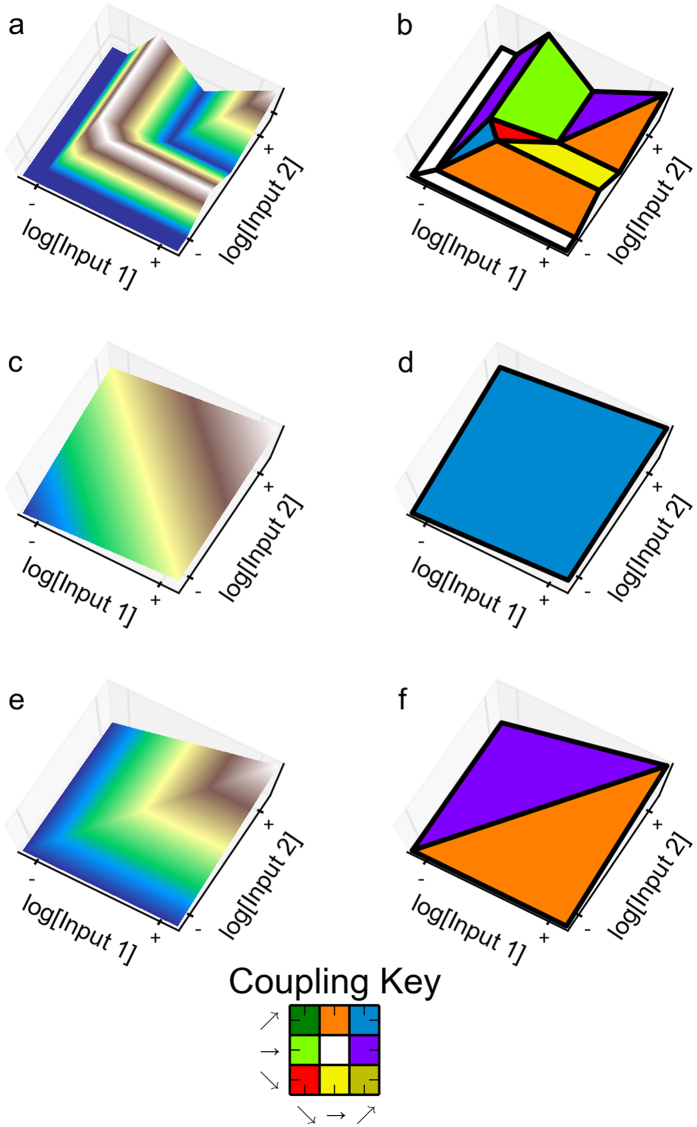
Decomposition of a landscape into unique basic landmarks. Panels on the left represent landscapes with the output value on the z-axis; brown and white colors represent values that are above the threshold, while all other colors represent values that are below the threshold. Panels on the right represent the deconstruction of the landscapes into landmarks with features represented by different colors as indicated by the key at the bottom: purple color represents direct coupling with input 1; orange represents direct coupling with input 2; blue represents direct coupling with both inputs. (**a,b**) Example of the deconstruction for a particular landscape reproducing an AND binary function into landmarks with various features. (**c,d**) Example of a basic landmark obtained by eliminating the white, red, yellow, green, purple and orange features of the non-basic landmarks in panel (**b**). (**e–f**) Example of a basic landmark obtained by eliminating the white, red, yellow, green and blue features of the non-basic landmarks in panel (**b**).

This reduction of landmarks to basic landmarks can be done iteratively by removing features until all remaining features are essential for reproducing the experimental observations. The complete set of basic landmarks for the realization of the experimental data together have all the features required of any landscape that reproduces the experimental observations. An example of a landscape with several landmarks and their decomposition into two basic landmarks for the AND gate is shown in Fig. 3.

#### Identifying sets of necessary local couplings

**Figure 4:**
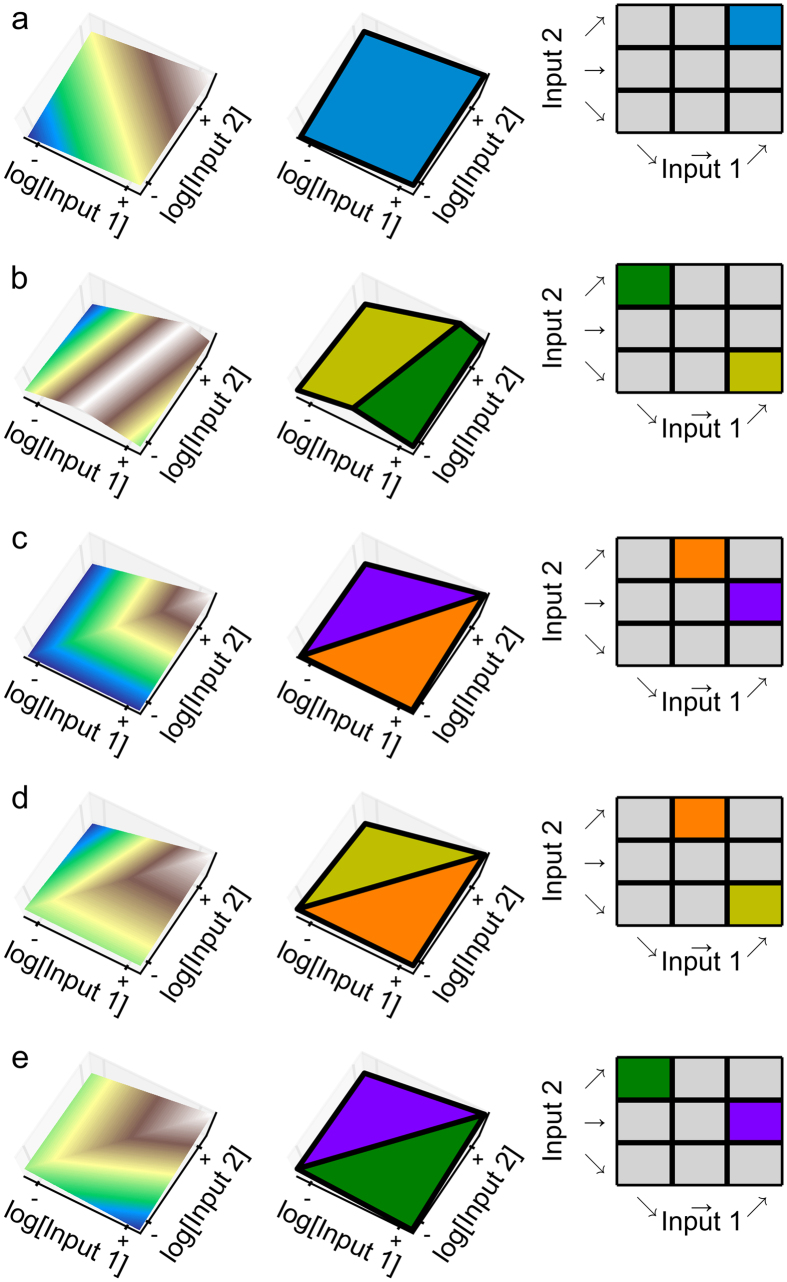
Basic landmarks, features and local couplings for the AND logic function. Left panels: Basic landmarks with the output value on the z-axis. The ‘−’ and ‘+’ symbols on the axes correspond to the low and high values of the experimental inputs, respectively. Center panels: Features on the z-axis corresponding to the panels on the left. Right panels: Local couplings between input and output are inversely coupled ↘, uncoupled →, and directly coupled ↗. Colored squares indicate the local couplings in the basic landmark as shown by the key in Fig. 3; gray squares represent local couplings that are not present in the basic landmark.

If all the basic landmarks necessary to reproduce the experimental observations have been identified, then the local couplings that are necessary also have been identified. Therefore, a model that cannot exhibit the local couplings for at least one of the basic landmarks cannot reproduce the experimental observations. It can be proved mathematically that the AND gate, with landmarks having strictly stable features, has only five basic landmarks [demonstrated in **Appendix**], shown in Fig. 4.

The set of five basic landmarks for the AND gate consists of one with a single feature and four with two features; in each case the features have a unique subset of local couplings. A particularly interesting basic landmark is shown in Fig. 4a, where the entire input variable space is a single feature. This implies that the feature in this basic landmark and its corresponding local coupling are able to represent the experimental data. The local coupling is a direct coupling between the two inputs and the output: if either input increases, the output also increases. Given this local coupling, a threshold value can be chosen such that the output will be above the threshold only when both inputs are high. Therefore, this local coupling is sufficient – at some scale – to represent the AND gate.

The remaining basic landmarks require at least two features with the following couplings: (1) the output is directly coupled to the first input and either inversely coupled or uncoupled to the second input, and (2) it is directly coupled to the second input and either inversely coupled or uncoupled to the first input. A graphical summary of the necessary local couplings for each of the basic landmarks is shown in the rightmost panels of Fig. 4.

These couplings are in general nonlinear, representing products and powers. Hence, they only appear to be linear because they are depicted in logarithmic coordinates. Thus, the output could be a steep nonlinear function of the inputs with a large Hill number. Moreover, the transition between the discrete data points observed experimentally need not involve graded incremental changes even if the underlying function is continuous. By continuing the analogy with geological landscapes, there may be a cliff that overhangs, and going over results in a precipitous drop to a new state. This is equivalent to a bifurcation where there is a transition involving an unstable state. We can extend the set of basic landmarks to include all those that traverse through regions of multi-stability involving a combination of stable and unstable features. We find that for each basic landmark involving stable features there is a corresponding basic landmark involving equivalent unstable features, as shown in Figure S1. In addition, for each basic landmark that involves 2 stable or 2 unstable features there is a basic landmark that involves one stable and one unstable feature. An example of two basic landmarks involving one stable and one unstable feature is shown in Figure S2. Taken together we find a total of 18 basic landmarks for an AND binary logic gate.

These results can be used to test multiple hypothesized mechanisms for their potential to realize an AND gate function, as we will show in the **Model-Discrimination Phase**. All other binary logic gate functions can be analyzed in the same way, and similar combinations of necessary local couplings can be identified.

### Model-Dependent Phase

The model-dependent phase is concerned with a quantitative description of a given model and the systematic deconstruction of its behavior into qualitatively-distinct phenotypes. This description is purely a function of the mechanisms proposed, and not a function of the discrete data observed experimentally. This description is obtained following a series of steps: (1) the model is described mathematically by a system of ordinary differential equations based on the underlying chemical and biochemical kinetics; (2) the model’s repertoire of qualitatively-distinct phenotypes is automatically identified using System Design Space methodology^12^; and (3) the local coupling is identified for each of its realizable phenotypes.

The computational workflow is as follows. (a) The mathematical model is recast exactly into the Generalized Mass Action (GMA) representation within the power-law formalism^17^. (b) The recast equations are used to construct all possible combinations of dominant sub-systems (S-Systems). (c) The steady-state solution for each dominant S-System is determined in logarithmic space (linear systems analysis). (d) The dominance conditions and the steady-state solution are combined to construct the boundary equations for each dominant S-System. (e) The region in parameter space determined by the boundary equations is tested for feasibility by using linear programming methodologies^18^,^19^. (f) If an S-System’s region is feasible, its local coupling is calculated using standard S-System analysis^20^, and it is added to the phenotypic repertoire in System Design Space. The model-dependent phase involves standard analysis using the System Design Space methodology that has been described in the literature^11^,^12^,^21^,^22^. Computations are carried out using open-source software tools^23^ based on an extension of the Design Space Toolbox in Matlab^®^^21^.

#### Quantitative mathematical models

In the context of biological systems composed of gene circuits, signal transduction cascades and biochemical networks, one typically starts with a conceptual model of the processes that bring about some observable or desired behavior. To analyze conceptual models we rely primarily on intuition and from these systems we obtain a qualitative understanding of the underlying phenomena. However, biological systems have nonlinear processes that are responsible for the emergence of complex phenotypes for which an intuitive level of understanding is inadequate; therefore, it becomes necessary to formulate mathematical models and apply rigorous quantitative methods to further understand these systems.

Our three-part strategy focuses on discriminating among hypotheses based on their inability to generate specific phenotypes. This class of systems involves mechanisms that are ultimately governed by chemical kinetics plus constraints. Therefore, the rates of synthesis and loss of biochemical species are typically described by deterministic rate laws, and mathematical models of these mechanisms are then integrated into systems of nonlinear differential (or differential algebraic) equations in the power-law formalism^17^.

A quantitative model for the transcriptional control of the *lacZYA* operon of *E. coli* will provide an illustration. This system has been shown to realize an AND logic function; thus, it will serve in the model-dependent phase as the counterpart of the example used in the model-independent phase of our strategy. This example has been selected because it is well established, it is simple enough for our intuition to be adequate, and there are experimental results that have already validated the model.

**Figure 5:**
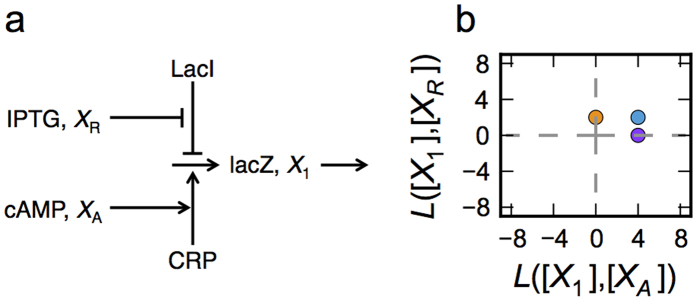
Control of *lacZ* transcription as an AND logic function. (**a**) Conceptual model representing control of *lacZ* by CRP activation and LacI repression. Barbed arrows represent a positive influence and blunt arrows represent a negative influence. (**b**) Local couplings of the mathematical model for control of *lacZ* transcription in Eq. (1). All the valid combinations of logarithmic gains in the phenotypic repertoire are represented as circles in this map. The color indicates the coupling that corresponds to those in the coupling key at the bottom of Fig. 3.

Setty *et al.*^3^ have shown that control of the *lacZYA* operon acts as an AND gate in which the two small molecule inducers cAMP and IPTG are the input signals and gene expression is the output signal. The currently accepted model for this control mechanism involves an upstream cis-regulatory region with binding sites for both cAMP Regulatory Protein (CRP) and *lac* repressor (LacI). CRP becomes active in the presence of cAMP and LacI becomes inactive in the presence of IPTG. The operon has a weak promoter that does not significantly initiate transcription unless CRP is present. However, if LacI is bound then transcription initiation is repressed even in the presence of CRP. Therefore, transcription initiation only occurs at a significant rate if both cAMP concentration is high, which results in CRP activation, and IPTG concentration is high, which results in LacI derepression, as shown schematically in Fig. 5a.

A mathematical model for promoter activity of the *lacZYA* operon can be readily formulated. Here, we use the existing model of Setty *et al.*^3^.


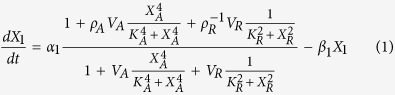


where *X*_1_ represents the concentration of *lacZYA* mRNA, *α*_1_ the basal rate of transcription, and *β*_1_ the rate constant for mRNA loss by inactivation/dilution. The output is identified with *X*_1_, and the inputs with *X*_*A*_, the concentration of cAMP, and *X*_*R*_, the concentration of IPTG. The following parameters associated with the activator, CRP, and the repressor, LacI, have subscripts *A* and *R*, respectively: *V*_*i*_ is the maximum rate of inducer-protein binding, *ρ*_*i*_ > 1 is the maximum capacity for regulation, and *K*_*i*_ the inducer concentration for half-maximal rate of inducer-regulator binding. It is important to stress that validation of this model has been achieved by determining specific values of the parameters that reproduce experimental results for the natural system. Here, we will provide a validation of this mathematical model as an AND gate in a manner that is exhaustive and independent of parameter values.

#### Deconstructing the model into sub-models with qualitatively distinct phenotypes

Models of genetic circuits, signal transduction cascades, and biochemical networks that adhere to chemical and biochemical kinetics can be recast exactly into the GMA form and analyzed analytically as well as computationally using the System Design Space methodology. A detailed mathematical treatment of the Design Space methodology is provided in the literature^11^,^21^,^22^. This methodology has been instrumental in elucidating the design principles of several natural systems (e.g. refs 24, 25, 26, 27, 28) and synthetic constructs (e.g. refs 5,12), and it is a critical component of our three-part strategy. It provides a mathematically rigorous definition of *phenotype*^11^,^12^ based on dominant processes operating within the integrated system. Having this definition allows for the deconstruction of complex systems into tractable sub-systems with qualitatively distinct phenotypes that are parallel to *local couplings* and *features* described in the Model-Independent Phase.

Here, we focus on two critical properties of the System Design Space methodology that will be essential for linking the Model-Dependent Phase and Model-Independent phase of our overall strategy. First, the System Design Space method decomposes a complex nonlinear system into tractable nonlinear sub-systems in the S-system form. Although the original system has a potentially *infinite* number of phenotypes, the System Design Space identifies a *finite* number of qualitatively-distinct phenotypes. Second, the method partitions logarithmic space into a space-filling set of *N*-dimensional convex polytopes, where *N* is the number of parameters and independent variables of the system. Each convex polytope defines the region within which a particular sub-system is a valid representation of the original system. Thus, each qualitatively-distinct phenotype has hyperplane boundaries in logarithmic space that are determined by solving a linear programming problem, which is a simple task amenable to well-known methods^18^,^19^. The collection of qualitatively-distinct phenotypes in System Design Space defines the *phenotypic repertoire* of the system; thus, this decomposition provides a tractable global characterization of the system^11^.

The first step in deconstructing the model in Eq. (1) is to recast it into the GMA form by defining auxiliary variables^11^,^12^,^22^. There are several ways to accomplish this, but a particularly simple way when dealing with rational functions is to define each denominator as a new variable. Let the complicated denominator be *X*_2_ and the two simpler denominators be *X*_3_ and *X*_4_. The result is the following set of differential algebraic equations in the GMA form.


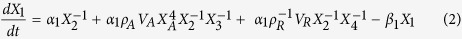



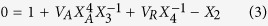



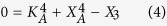



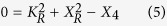


This system may now be analyzed using the System Design Space methodology. Inspection of Eqs (2, 3, 4, 5) reveals that there are 3 possible dominant terms of a given sign in Eq. (2), 3 in Eq. (3), 2 in Eq. (4), and 2 in Eq. (5). Together, it yields a total of 36 possible combinations of dominant terms. However, not all combinations are valid and thus we apply linear programming techniques to determine those that are feasible and correspond to qualitatively distinct phenotypes somewhere in parameter space. Analysis of the recast system in Eqs (2, 3, 4, 5) yields the phenotypic repertoire of the system, which involves 19 qualitatively distinct phenotypes.

#### Local coupling in the phenotypic repertoire

The phenotypic repertoire of the system is a collection of dominant subsystems represented by S-system equations. These S-system equations are nonlinear yet they are analytically tractable for determining properties such as steady-state concentrations and fluxes, logarithmic gain factors for signal propagation, parameter sensitivities for local robustness, amongst many others^11^,^20^,^21^,^29^. In the context of this paper, we deal with changes in the output signal as a function of the input signals and hence the local couplings of interest are the logarithmic gains^20^. The logarithmic gain is defined as the percentage change in an output variable with respect to the percentage change in steady state of an input variable, with other input variables held constant,


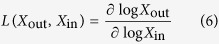


S-system models have logarithmic gains that are solely a function of the kinetic orders and, since these are chemical parameters with fixed values in the rational functions of biochemical kinetics and hence are architectural features of any model, logarithmic gains are constants that define linear gradients in logarithmic space throughout an entire phenotypic region. The qualitative values of the logarithmic gains represent the three possibilities for coupling between an input and output: directly coupled L > 0, inversely coupled L < 0, and uncoupled L = 0. For the specific case of two inputs and a single output there are two logarithmic gains, and the relevant local coupling will have one of 3 × 3 = 9 possible values.

As we described in the Model-Independent Phase, the stability of the fixed points is also important and must also be considered in the Model-Dependent Phase. As was the case with the logarithmic gains, S-System models have exponential instabilities that are solely a function of the kinetic orders (where the S-System has one eigenvalue with positive real part). Thus, if an S-System model has one eigenvalue with positive real part, its value is constant and the phenotype is exponentially unstable throughout the entire phenotypic region.

The logarithmic gains for a particular phenotype of the system can be represented as a dot in a two dimensional plot, where the *x-axis* is the logarithmic gain of the output with respect to the first input signal, and the *y-axis* is the logarithmic gain of the output with respect to the second input signal. A scatter plot represents the set of local couplings of the entire phenotypic repertoire, where each phenotype of the system is represented with a dot in this two-dimensional space. The stability of a phenotype is represented by the shape of the dot (e.g., circles represent stable phenotypes; pentagons represent unstable phenotypes).

In the previous section we identified a total of 19 valid phenotypes for the model of *lacZYA* transcription, and we can represent each phenotype by a point in the scatter plot of log-gains as shown in Fig. 5b, where the output signal is *lacZYA* mRNA levels, and the input signals are concentrations of cAMP and IPTG. The relevant phenotypes of the repertoire can be further selected by adding bounds to restrict values of the parameters to biologically relevant ranges. For the scatter plot in Fig. 5b, we have added the architectural constraint on the capacity for regulation, given by *ρ*_*i*_ > 1, of both activator and repressor such that the modes of regulation are conserved.

### Model-Discrimination Phase

The model-discrimination phase involves a direct comparison of the results from the model-independent and model-dependent phases using a step-wise series of tests performed at two levels, the architectural level and the biological level.

#### General strategy for model discrimination

Discrimination at the architectural level involves determining whether or not the discrete experimental data are reproduced by a model without imposing constraints on the values of its parameters, as long as the system architecture is conserved. In contrast, discrimination at the biological level involves determining whether or not the discrete experimental data are reproduced with biologically feasible values for its parameters. In the context of our analysis, discrimination at the biological level typically involves restricting parameter values to a range about their experimentally measured or estimated values. The following step-wise series of tests makes heavy use of our automated System Design Space strategy^12^ by (a) identifying a set of parameter values for the realization and characterization of each qualitatively-distinct phenotype, (b) identifying an *n*-dimensional slice of System Design Space that allows simultaneous realization of several phenotypes, and (c) identifying an arrangement of phenotypes capable of modeling specific characteristics to achieve desired functions (e.g. binary logic functions).

The steps involve determining whether (1) a model has the necessary logarithmic gains consistent with at least one set of local couplings necessary to generate a basic landmark for a given set of experimental data; (2) there are phenotypes from Step (1) that can be simultaneously realized within a particular slice of Design Space, where the slice variables are the input parameters, by applying our automated System Design Space methods^12^; (3) there are simultaneously realized phenotypes from Step (2) that can be arranged to yield the appropriate logic function with the appropriate output profile by choosing an arbitrary threshold; and (4) if a hypothesis involves determining whether models for multiple systems satisfy different sets of observations under the same experimental conditions, we can repeat Step (3) for all systems simultaneously to determine if there is a common set of values for the parameters that can explain all logic functions across multiple systems. This 4-step approach can be applied to determine whether models have the potential to account for binary logic functions at either the architectural level, or at the biological level by choosing to add constraints on the permissible values for the parameters^12^.

There are several possible outcomes: (1) If the hypothesized model is unable to reproduce the set of local couplings for any of the basic landmarks, then it fails to satisfy the necessary conditions to reproduce the observed data; therefore the model is discarded. (2) If the model can reproduce the set of local couplings for at least one basic landmark, then it satisfies a necessary condition but sufficiency must still be determined. (3) If the model exhibits a local coupling corresponding to a single-feature basic landmark, then the local coupling is capable of reproducing the experimental data at some scale and thus is sufficient. (4) If the model exhibits a combination of local couplings corresponding to a multi-feature basic landmark (e.g. all but the basic landmarks in Fig. 4a), then we must determine whether or not the qualitatively distinct phenotypes with these local couplings can be simultaneously realized. Furthermore, we must determine if they are positioned appropriately such that the correct binary logic function is generated. This task is not trivial given that mechanistic models often involve a large number of parameters. However, our System Design Space methods can automatically determine whether a set of qualitatively distinct phenotypes can be simultaneously realized in specific arrangements, without sampling parameter values^12^. (5) If a hypothesis satisfies the conditions from the model-dependent phase at the architectural level, then we can perform all the previous tests at the biological level. (6) If a model has passed these tests at the biological level, then we can automatically identify sets of parameter values that generate the desired ensembles of phenotypes^12^.

#### Model discrimination in the lacZYA case

The model discrimination phase in this case can be performed through direct visual comparison. By revisiting Fig. 5b, we see that all the phenotypes in this model are stable, thus we only need to compare the right-most panels of Figs 4 and 5b. The necessary condition states that the local couplings of the phenotypic repertoire, shown in Fig. 5b, must be consistent with the local couplings for at least one basic landmark associated with the experimental observations, shown in Fig. 4. Inspection of Fig. 5b reveals that the phenotypic repertoire has the local couplings required for three basic landmarks identified in Fig. 4a–c. Hence, the model satisfies the necessary conditions for the experimental observations.

Although this model exhibits the necessary local coupling for multiple basic landmarks, we will focus on the plane discriminator in Fig. 4a, where the output signal is directly coupled to both input signals. Thus, with this focus on the plane discriminator, the model satisfies not only necessary but also sufficient conditions at the architectural level and thus can reproduce the observed experimental data somewhere in parameter space, at some scale. Previous analysis of this model had shown that it could reproduce experimental observations exhibiting AND logic by providing a direct example with specific parameter values^3^. Our analysis also has shown that the model can reproduce the appropriate experimental observations; however, our analysis has done so in a manner that is independent of parameter values. Furthermore, our method can automatically determine sets of parameter values that yield the AND logic function, and the parameter values can be constrained to biological ranges^12^.

## Application to Synthetic Constructs Exhibiting Unexpected Behaviors

We illustrate our strategy by focusing on two experimentally synthesized genetic constructs, the D038 and D052 constructs, reported in the literature^7^. These constructs involve some of the best-characterized transcriptional repressors, LacI, TetR and λ CI, coupled to control the expression of a reporter gene, *gfp*. The promoter elements in the D038 and D052 constructs are the same with the exception of the target of LacI.

### Conventional Models

**Figure 6:**
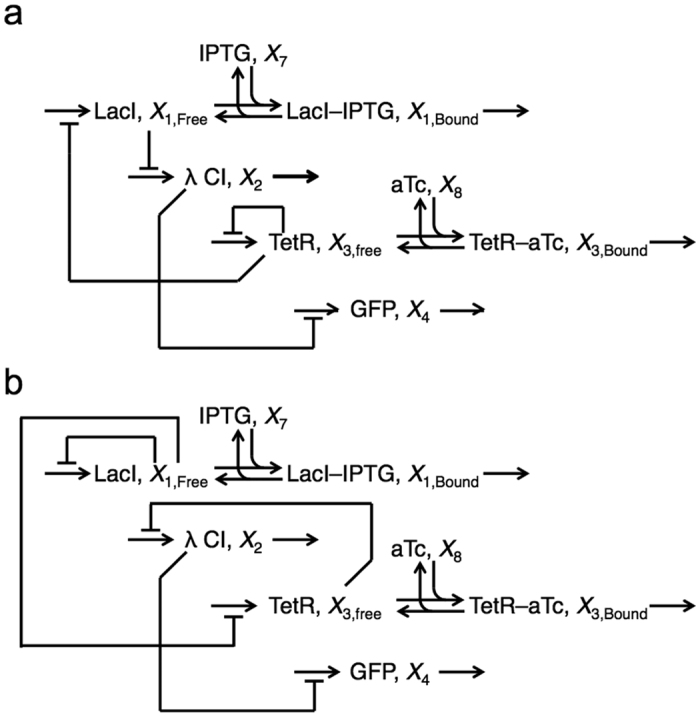
Conceptual models for experimental constructs consisting of a three-gene hierarchical regulatory circuit. Transcription and translation are collapsed into a single kinetic-step, without loss of generality in steady state. The ordering of genes represents the order in which they are encoded on the plasmids constructed by Guet *et al.*^7^. (**a**) The TetR-primary circuit and (**b**) the LacI-primary circuit.

The LacI repressor undergoes a conformational shift when bound to the small molecule inducer isopropyl β-D-1 thiogalactopyranoside (IPTG). Similarly, the TetR repressor undergoes a conformational shift when bound to anhydrotetracycline (aTc). This change in structure reduces the binding affinity for specific DNA sequences within the promoter regions of their target genes, thus relieving repression and causing an induction of target gene expression^30^,^31^. The repressor λ CI does not respond to either of these gratuitous inducers that constitute the environmental signals used in the reported experiments^7^. These transcriptional circuits are represented by the diagrams in Fig. 6.

#### Circuit architecture and expected results

The transcriptional circuits, represented by the diagrams in Fig. 6, share a general topology (an architectural feature) in which the transcriptional repressors are coupled to form a hierarchical regulatory cascade involving an inducible primary repressor, an inducible secondary repressor, a tertiary repressor, and a reporter protein. The primary repressor protein binds to the promoter region of its own gene and that of the gene for the secondary repressor, thus repressing gene expression of both the primary and secondary repressors. The secondary repressor binds to the promoter region of the gene for the tertiary repressor to cause its repression. The tertiary repressor binds to the promoter region of the gene encoding a fluorescent reporter protein, thus repressing gene expression and consequently cell fluorescence.

#### Circuit architecture

Although the general topology is the same for both circuits, the primary and secondary repressors are switched such that the primary repressor in one circuit is the secondary repressor in the other and vice versa. We refer to the individual circuits by their primary repressor; hence, the gene circuit in Fig. 6a is the TetR-primary circuit and that in Fig. 6b is the LacI-primary circuit.

Note that the D038 and D052 constructs depicted in Fig. 2 of Guet *et al.*^7^ share the same promoters, with exception of the *lac* promoter for which two are shown. However, they also report experimental data showing that the qualitative aspects of the D052 construct are the same, regardless of the specific *lac* promoter used^7^ Here, we assume that the *lac* promoters are identical, since this represents a more stringent comparison.

#### Experimental observations

The binary logic functions reported by Guet *et al.*^7^ are ‘fuzzy’ as they depend on an arbitrary fluorescence threshold to discriminate between the binary output states. Here, we reevaluate the observed fluorescence profiles and focus on the qualitative nature of the results to identify the appropriate binary logic functions that will be the focus of our analysis.

The qualitative nature of the published results is as follows. The TetR-primary circuit, shown in Fig. 6a, exhibits high fluorescence intensity at a low IPTG and high aTc (−/+); background level fluorescence at high IPTG and high aTc (+/+); and low fluorescence at the remaining conditions (+/− and −/−). In contrast, the LacI-primary circuit, shown in Fig. 6b, exhibits high fluorescence intensity at low IPTG and low aTc (−/−); and background level fluorescence intensity at all other experimental conditions (+/−, −/+, +/+) [data reported from Fig. 6A in Guet *et al.*^7^]. The reported result for the TetR-primary circuit is a conditional “aTc AND NOT IPTG”, also known as a NIF1, binary logic function. A fluorescence intensity threshold can be chosen such that the conditional logic function is produced; however, an alternative threshold can be chosen such that the construct exhibits a NOT AND, also known as a NAND, binary logic function. Therefore, the TetR-primary circuit must be capable of generating both NIF1 and NAND logic depending on the chosen fluorescence threshold. In the context of our analysis, the global results we assume are the following: the TetR-primary circuit generates a NAND logic gate at a low threshold and a NIF1 logic gate at a higher threshold; and the LacI-primary circuit generates a NOT OR, also known as a NOR, logic gate.

Close inspection of the data presented by Guet *et al*, specifically their Fig. 2, shows that the NAND gate behavior is not restricted to the lac+ strain, but is in fact observable in the lac- strain as well. This effect, albeit marginal, can be seen from the distribution of cells by fluorescence intensity, where the cells in the +/+ state are on average biased towards a lower fluorescence intensity when compared to the cells in the −/− and +/− states. This is most noticeable by comparing the peak fluorescence intensities. Furthermore, visual inspection of the plates, also shown in their Fig. 2, reinforces the conclusion that there is a slight difference in fluorescence between the aforementioned states. Thus, we will focus on the results for the lac- strain because this represents a more stringent test for our methods.

#### Intuitive analysis of the conventional models

If we were to consider the results for these circuits intuitively, based solely on the network architecture, we would expect distinct logic functions based on what we have described above. In the case of the TetR-primary circuit, a high concentration of IPTG would lead to derepression of λ CI expression, which would result in an ‘OFF’ state regardless of the concentration of aTc. Hence, a NIF1 logic gate would be expected. The opposite should be true for the LacI-primary circuit, a high concentration of aTc would lead to derepression of λ CI expression, which would in turn repress GFP expression and hence translate into an ‘OFF’ state regardless of the concentration of IPTG. Therefore, an “IPTG AND NOT aTc”, also known as a NIF2, logic gate would be expected. This analysis suggests that the physical constructs are functioning in a manner that is counterintuitive and *anomalous*. However, as with any complex system, our intuition is likely to fail us and a more rigorous analysis of these systems is called for.

#### Model-Independent Phase: NAND, NOR and NIF1 logic gates

The model-independent phase, reiterated briefly here, consists of the following series of steps: we (a) propose sets of continuous functions that generate *landscapes* consisting of *landmarks* that can account for the observed data, (b) identify a set of landmarks, called *basic landmarks*, that are the kernels of all possible landscapes, (c) identify *features* of the basic landmarks that have a qualitatively distinct input-output coupling and (d) identify the necessary sets of input-output couplings, or *local couplings*, within the features.

**Figure 7:**
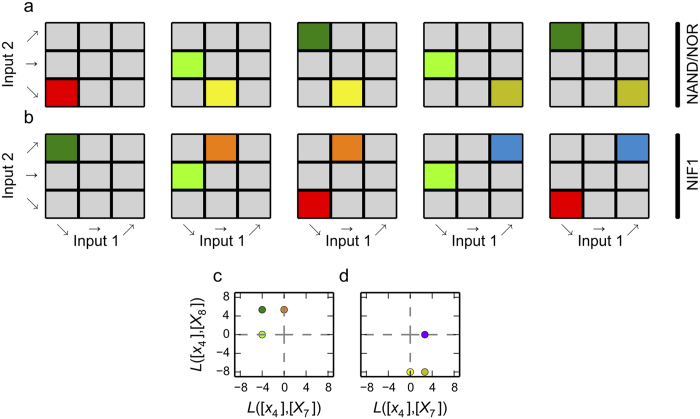
Graphical summary of the three-phase analysis of the conventional models for the LacI-primary and TetR-primary gene circuits. (**a,b**) Graphical representation of the necessary local couplings for the basic landmarks obtained from the model-independent phase of the analysis. The arrows on the x- and y-axes represent the coupling between input and output: Inversely coupled ↘, uncoupled →, and directly coupled ↗. Each grid of 3 × 3 squares shows a set of local couplings for the features of a particular basic landmark (indicated by the colored squares). Necessary coupling for basic landmarks of (**a**) NAND and NOR logic gates and (**b**) the NIF1 logic gate. (**c,d**) Graphical representation of the repertoire of qualitatively distinct phenotypes from the model-dependent phase of analysis for the (**c**) TetR-primary and (**d**) LacI-primary circuits. Each circle represents one or more qualitatively distinct phenotypes with a unique combination of logarithmic gains (relative derivatives of the output, *X*_4_, with respect to the inputs, *X*_7_ and *X*_8_). The colors represent the type of coupling as shown in panels (**a**) and (**b**) and Fig. 4.

In order to determine whether the conventional models of the TetR-primary and LacI-primary circuits are consistent with the experimental observations, we identified all the basic landmarks for the NAND, NOR and NIF1 binary logic functions. We found five basic landmarks for each assuming all features are stable. For each of these basic landmarks, we identified the necessary sets of local coupling, represented visually in Fig. 7a,b. Alternatively, there are a total of 18 basic landmarks if we allow for unstable features in the transition between the experimental observations (data not shown).

#### Model-Dependent Phase

In the following sub-sections we describe the details of the model-dependent phase, applied to the conventional circuits for the experimentally reported constructs. This phase consists of (a) proposing a mathematical model that represents the hypothesis being tested, (b) applying the System Design Space method to determine the model’s repertoire of qualitatively distinct phenotypes^11^,^12^,^21^ and (c) identifying the log-gain functions, a quantitative measure of the input-output coupling.

#### Model formulation

The constructs reported in the literature have been synthesized in a linear sequence on a plasmid using a combinatorial approach, where the promoter regions of the *lacI*, λ *cI* and *tetR* genes were allowed to have any of five different promoters^7^. Here, we formulate mathematical models for two of the circuits that were experimentally characterized in detail.

The models, composed of ordinary differential equations (ODEs), assume that the turnover of mRNA is fast relative to that of protein and that the concentration of protein directly tracks that of the mRNA, the conventional assumption for many models in synthetic biology. Thus, we model the modulation of transcription as having a direct effect on the rate of protein synthesis. The generic equations that describe the gene circuits are then


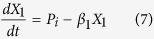



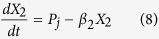



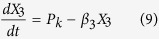



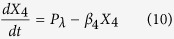


where *X*_1_ represents the total concentration of LacI (tetramers), *X*_2_ represents the total concentration of λ CI (dimers), *X*_3_ represents the total concentration of TetR (dimers), and *X*_4_ represents the total concentration of GFP. The numbering of the variables reflects the order in which the genes appear encoded in the plasmid DNA.

The rates of synthesis reflecting transcription from promoters repressed by LacI, TetR and λ CI are represented by the following rational functions.


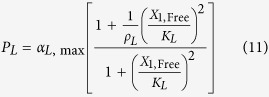



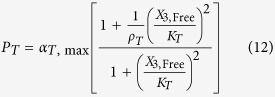



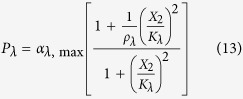


where subscripts *L*, *T* and *λ* correspond to parameters associated with the *lac*, *tet*, and *λ* promoters; the *α*_*j*, max_ parameters represent the maximal rate of protein synthesis, where *j* = {*L*, *T*, *λ*} represents the regulator controlling promoter activity; the parameters *ρ*_*j*_ > 1 represent the capacity for transcriptional regulation – defined as the ratio of maximal to basal rate of transcription; the parameters *K*_*j*_ represent the concentration of regulator for half-maximal influence on the rate of transcription; and the repressors are assumed to bind as dimers (dimer of dimers for LacI).

The variables *X*_1,Free_ and *X*_3,Free_ represent the concentrations of free LacI and free TetR, which are obtained by assuming a quasi-steady state for repressor-inducer binding,


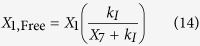



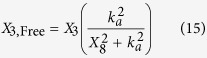


The symbols *X*_7_ and *X*_8_ represent the concentration of IPTG and aTc, *k*_*I*_ and *k*_*a*_ represent the inducer concentration for half-maximal rate of binding between LacI with IPTG and TetR with aTc; it is assumed that LacI (dimer) and TetR (monomer) are inactivated by the binding of one and two molecules of inducer^32^.

We focus on the TetR-primary and LacI-primary circuits, which correspond to the D038 and D052 constructs of Guet *et al*^7^. These circuits are defined by *P*_*i*_ = *P*_*T*_*, P*_*j*_ = *P*_*L*_, *P*_*k*_ = *P*_*T*_ for the TetR-primary circuit and *P*_*i*_ = *P*_*L*_*, P*_*j*_ = *P*_*T*_, *P*_*k*_ = *P*_*L*_ for the LacI-primary circuit. Thus, the mathematical model for the TetR-primary circuit is defined by the system of Eqs (7) to (10), following the substitution of *P*_*T*_ from Eq. (12) for *P*_*i*_ and *P*_*k*_ in Eqs (7) and (9) and the substitution of *P*_*L*_ from Eq. (11) for *P*_*j*_ in Eq. (8). The LacI-primary circuit is defined by the system of Eqs (7) to (10) following the substitution of *P*_*L*_ from Eq. (11) for *P*_*i*_ and *P*_*k*_ in Eqs (7) and (9) and the substitution of *P*_*T*_ from Eq. (12) for *P*_*j*_ in Eq. (8).

#### Analysis

The mathematical equations for the TetR-primary and the LacI-primary circuits were analyzed using the same approach used for the *lacZYA* operon. For each circuit, the total number of combinations of dominant positive and dominant negative terms is 256, which defines the maximal number of sub-systems and hence the potential number of qualitatively distinct phenotypes for each circuit^11^,^12^. Of the 256 potential phenotypes, only 108 are valid somewhere in parameter space, all of which involve stable fixed points, and these constitute the phenotypic repertoire for each circuit. The valid sub-systems were analyzed for their logarithmic gains, *L*([GFP], [IPTG] and *L*([GFP], [aTc], to identify the local couplings shown graphically in Fig. 7c,d.

#### Model-Discrimination Phase

The model-discrimination phase of our strategy involves a series of tests using the results of the previous two phases. We applied our 4-step approach described for the general case to the conventional models of the TetR-primary and LacI-primary circuits. The details of each step are outlined below,

#### Step 1: Determine whether the models have the necessary local couplings in the phenotypic repertoire

In this step, we group phenotypes of interest into 2 sets for the LacI-primary circuit and 3 sets for the TetR-primary circuit.

For the LacI-primary circuit, with the observed NOR binary logic function, we group phenotypes into the following sets according to their coupling and stability features:

Set 1: {↘, →[Stable]; ↘, ↗[Stable], ↘, ↘[Stable]; ↗, →[Unstable]; ↗, ↘ [Unstable]; ↗, ↗ [Unstable]}

Set 2: {→, ↘ [Stable]; ↗, ↘ [Stable]; ↘, ↘ [Stable]; →, ↗ [Unstable]; ↘, ↗ [Unstable]; ↗, ↗ [Unstable]}

where the first arrow represents the coupling of GFP with respect to IPTG, the second arrow represents the coupling of GFP with respect to aTc, and the stability of the fixed points for the phenotypes is shown in square brackets.

For the TetR-primary circuit, with the observed NAND and NIF1 binary logic functions, we group phenotypes into the following sets according to their coupling and stability features:

Set 1: {→, ↘ [Stable]; ↗, ↘ [Stable]; ↘, ↘ [Stable]; →, ↗ [Unstable]; ↘, ↗ [Unstable]; ↗, ↗ [Unstable]}

Set 2: {↘, →[Stable]; ↘, ↗[Stable], ↘, ↘[Stable]; ↗, →[Unstable]; ↗, ↘ [Unstable]; ↗, ↗ [Unstable]}

Set 3: {→, ↗ [Stable]; ↗, ↗ [Stable]; ↘, ↗ [Stable]; →, ↘ [Unstable]; ↘, ↘ [Unstable]; ↗, ↘ [Unstable]}

The basic landmarks for their respective logic functions require that each model have at least one phenotype in each of their corresponding sets. Note that the basic landmarks for NAND and NOR binary functions involve the same features; however, their relative arrangements are inverted to generate the opposite binary logic functions. Therefore, Set 1 and Set 2 for the LacI-primary and TetR-primary circuits are switched at this point, which simplifies later stages of the analysis.

#### Step 2: Determine whether phenotypes can be simultaneously realized

This step involves determining whether there is at least one combination involving one phenotype from each of the sets defined in Step 1 that can be simultaneously realized. We apply our previously developed methods for simultaneously realizing qualitatively distinct phenotypes (formally called phenotype co-localization)^12^. Specifically, this method automatically identifies a set of parameter values, if it exists, such that qualitatively distinct phenotypes are ‘found’ within an *n* dimensional slice of Design Space (in this example, we are looking at a 2-D slice where the slice variables on the axes are the concentrations of IPTG and aTc). It is important to note that the parameter values are not manually picked, sampled or estimated from the data; instead, our software automatically predicts the parameter sets by applying techniques from linear programming and optimization. As such, the parameter sets predicted by our method are not unique, but they are representative of points within a high-dimensional polytope in logarithmic coordinates. Details are provided elsewhere^12^.

#### Step 3: Determine whether co-localized phenotypes can be arranged to give the correct logic function with the appropriate output profile by choosing an arbitrary threshold

This step involves determining whether combinations of phenotypes that can be simultaneously realized, as determined in Step 2, are realized in the appropriate arrangement. The arrangement is determined by repeating the co-localization described briefly for Step 2 with additional constraints on auxiliary variables representing the slice variables for each phenotype.

For the LacI-primary circuit, with the observed NOR binary logic function, the phenotype in Set 1 must be localized to the bottom and right of the phenotype in Set 2. Therefore, we add the following constraints,


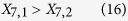



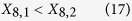


where *X*_7,*i*_ corresponds to an auxiliary variable representing *X*_7_ for the phenotype in the *i*-th Set, and *X*_8,*i*_ corresponds to an auxiliary variable representing *X*_8_ for the phenotype in the *i*-th Set. In addition, for each phenotype we impose the constraint


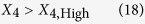


where *X*_4,High_ is a variable representing the threshold concentration for an ‘On’ state.

For the TetR-primary circuit, with the observed NAND and NIF1 binary logic functions, the phenotypes in Set 1 must be localized to the bottom and right of the phenotypes in Set 2 (these were swapped compared to the sets for the LacI-primary circuit). In addition, the phenotype in Set 3 must be localized to the top and left of the phenotype in Set 1 and to the top and right of the phenotype in Set 2. Therefore, we add the following constraints,


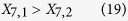



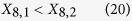



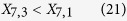



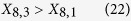



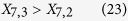



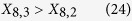


where *X*_7,*i*_ corresponds to an auxiliary variable representing *X*_7_ for the phenotype in the *i*-th Set, and *X*_8,*i*_ corresponds to an auxiliary variable representing *X*_8_ for the phenotype in the *i*-th Set. In addition, we impose the following constraints on the concentration of each phenotype,


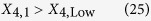



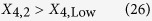



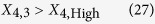


where *X*_4,High_ is a variable representing the threshold concentration for the ‘On’ state and *X*_4,Low_ is a variable representing the threshold concentration for the ‘Intermediate’ state, such that


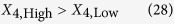


#### Step 4: Determine whether Step 3 can be repeated simultaneously for both the TetR-primary and LacI-primary circuits with a shared set of values for their parameters

This step involves determining if combinations of phenotypes that could be arranged correctly, as determined in Step 3, can be done so simultaneously for the models of both the TetR-primary and the LacI-primary circuits. This involves identifying a single set of values for their parameters such that the phenotypes in Set 1 and 2 of the LacI-primary circuit and in Sets 1, 2 and 3 of the TetR-primary circuit are co-localized in a single 2-D slice of Design Space. If both models have phenotypes that can be arranged simultaneously, we state that a hypothesis satisfies the necessary conditions to realize the observed binary logic functions for both circuits.

#### Comparison of phenotypic repertoires and necessary basic landmarks

When the 4-step approach is applied to the conventional models of the TetR-primary and LacI-primary circuits, we find that both models fail at the first step: the phenotypic repertoire of neither model included phenotypes with the local coupling needed to reproduce even a single basic landmark, as shown in Fig. 7.

The first test is to ascertain if the model has the necessary phenotypes to reproduce a basic landmark for the appropriate logic gate. The TetR-primary circuit, which only has stable phenotypes, has the necessary phenotypes for two basic landmarks of a NIF1 logic function, as can be seen by comparing Fig. 7c with the first two panels in Fig. 7b. Furthermore, the point in the top-left quadrant of Fig. 7c is consistent with the necessary local coupling for the first basic landmark in Fig. 7b. This basic landmark involves a single feature that spans the entire space, thus the local coupling produces a plane discriminator capable of exhibiting the NIF1 logic function. Therefore, this circuit is capable of exhibiting the NIF1 logic somewhere in parameter space.

However, neither the TetR-primary nor the LacI-primary circuit has a phenotype that falls within the lower-left quadrant, as shown in Fig. 3c,d; thus, neither circuit can produce the first basic landmark in Fig. 7a. The remaining basic landmarks in Fig. 7a require a point that lies within the top-left quadrant and a point within the bottom-right quadrant in Fig. 3c,d. In both cases, the circuits are capable of exhibiting phenotypes that satisfy one of these two conditions, but not both; hence, neither circuit can produce a basic landmark associated with either a NAND or a NOR binary logic function. These results, taken together, demonstrate that neither circuit can exhibit the observed experimental data. In other words, the conventional models of the synthetic constructs cannot –regardless of values for the parameters – exhibit the experimentally observed results.

### Single Mechanistic Extensions of the Conventional Models

Our analysis in the previous section has shown that the conventional models for the TetR-primary and the LacI-primary circuits cannot reproduce the experimentally observed results. This suggests that additional mechanisms are significantly influencing the physical constructs. Here, we focus on known mechanisms, many of which have been studied for many decades: Transcriptional read-through, specific binding of the LacI-IPTG complex to operator DNA, non-specific binding of LacI to backbone DNA (independent of IPTG), and regulator specific cross-talk, where one regulator binds to degenerate binding sites in one of the promoters that is regulated by a different regulator – thus repressing transcription of non-target genes.

It has been shown that transcriptional read-through and LacI-inducer complexes can play important roles in affecting gene regulation^33^,^34^,^35^. As discussed previously, the binding of LacI with IPTG causes a conformational change that affects the specific DNA binding domain of the protein. However, the change in structure does not abolish specific binding to the operator DNA, but reduces it by about 1000-fold^34^. Additionally, the LacI protein has a non-specific binding component that is thought to be involved in ‘sliding’ along the DNA chain, thus improving the efficiency of locating operator DNA. This non-specific binding occurs between LacI and the DNA backbone and has a 10^5^- to 10^10^-fold lower affinity relative to that of the specific binding^35^. However, this non-specific binding appears to be unaffected by binding of LacI with IPTG^33^ and hence, at high concentrations of LacI, there could be a significant influence from non-specific repression.

Furthermore, transcription factors bind a set of degenerate sequences with varying affinities^36^. Such binding sites for the LacI, TetR or λ CI regulators may be present upstream of any gene in the plasmid, thus adding ‘cross-talk’ where one regulator represses expression of non-target genes. Here, we consider the effect of cross-talk by each of the regulators over each of the promoters as a unique hypothesis.

Many of these mechanisms have been studied in the context of the natural *lac* operon, and are routinely assumed to be negligible within that context. However, the genetic components in the synthetic constructs are no longer operating in their native context and, in particular, LacI in the synthetic constructs may be induced to concentrations much greater than the low level constitutive expression in the native context. This begs the question, are the native assumptions still valid?

We extended the conventional mathematical models for the LacI-primary and TetR-primary circuits to include a total of 9 alternative mechanisms. The extended models serve as hypotheses that can aid in understanding the possible causes for the discrepancies between the observed results and the conventional models. It is important to note that although the LacI-primary and TetR-primary circuits have the same topology in the conventional models, this is no longer true in the mechanism-extended models. For example, consider the extension where TetR represses expression of P^L^. The TetR-primary circuit has one additional interaction – TetR also represses lambda transcription. The LacI-primary circuit has two additional interactions – TetR represses lacI transcription and autorepresses its own transcription.

#### Mathematical models of the mechanism-extended circuits

The following sub-sections provide mathematical descriptions for distinct mechanisms by which the conventional circuitry can be extended: (a) transcriptional read-through, (b) LacI-IPTG specific repression, (c) LacI non-specific repression, (d) LacI repression of the TetR target promoter, (e) LacI repression of the λ target promoter, (f) TetR repression of the LacI target promoter, (g) TetR repression of the λ target promoter, (h) λ CI repression of the LacI target promoter, and (i) λ CI repression of the TetR target promoter.

#### Transcriptional read-through

Transcriptional read-through involves a small probability of upstream transcription continuing past a transcriptional terminator and hence transcribing downstream genes. The percentage of transcription that reads-through is a function of the efficiency of the transcriptional terminator, and is represented in the following equations.


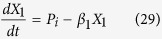



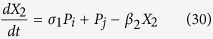



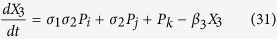



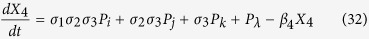


where the *σ*_*i*_parameters represent the probability of reading-through the *i*^th^ promoter. This model assumes that the ribosome binding sites are the same for all genes and is thus consistent with the physical constructs reported in the literature.

#### LacI-IPTG specific repression

LacI-IPTG specific repression involves specific operator binding by the LacI-IPTG complex. This mechanism is represented mathematically by adding a new term in the numerator and denominator of Eq. (11), which yields


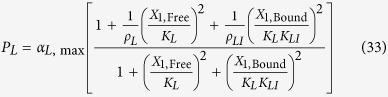


where *K*_*LI*_ > 1 represents the ratio of binding constants for the bound and free forms of the LacI regulator; *ρ*_*LI*_ >1 is the capacity for regulation by the LacI-IPTG complex. The amount of complex, *X*_1,Bound_, is defined by


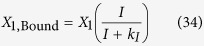


such that *X*_1_ = *X*_1,Free_ + *X*_1,Bound_.

#### LacI non-specific repression

LacI non-specific repression involves non-specific binding of LacI to backbone DNA. This mechanism is represented mathematically by adding a term to the numerator and denominator of the expressions representing transcription from the *lac*, *tet*, and *λ* promoters. The mathematical expressions representing the rate of transcription from these promoters become


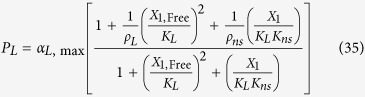



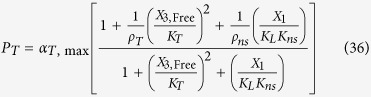



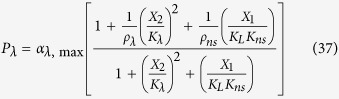


where *K*_*ns*_ > 1 × 10^5^ is the ratio of non-specific to specific binding constants for the LacI regulator; *ρ*_*ns*_ > 1 is the capacity for regulation by LacI non-specific binding, which is assumed to bind non-cooperatively as a dimer.

#### Regulator specific cross-talk

Cross-talk is defined as the repression of non-target promoters by a transcriptional regulator binding to unidentified sequences. Here, we consider that cross-talk is possible by any of the three regulators: LacI, TetR and *λ* CI. We model each cross-talk hypothesis individually to identify critical cross-talk interactions contributing to the observed experimental data. These hypotheses involve the following core assumptions: (1) non-target promoter binding sites are found within the promoter regions; (2) regulator binding to non-target promoters occurs with lower affinity than that of binding sites in the target promoter; (3) repression of a promoter by targeted and non-targeted regulators occurs independently; and (4) non-target binding involves a single binding site and thus is non-cooperative. These mechanisms are represented mathematically by adding a term to the numerator and denominator of the expression representing transcription from the target promoter.

LacI repression of the *tet* promoter is represented mathematically as


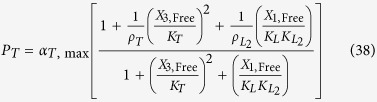


where 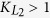 is the ratio of the binding constant for the non-target *tet* promoter binding site to the binding constant for the specific binding site for the LacI regulator; 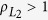 represents the capacity for regulation of the *tet* promoter by LacI repressor.

LacI repression of the λ promoter is represented mathematically as


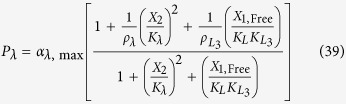


where 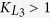 is the ratio of the binding constant for the non-target *λ* promoter binding site to the binding constant for the specific binding site for the LacI regulator; 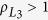 represents the capacity for regulation of the *λ* promoter by LacI repressor.

TetR repression of the *lac* promoter is represented mathematically as


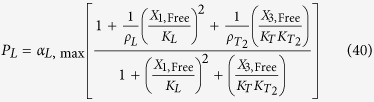


where 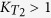 is the ratio of the binding constant for the non-target *lac* promoter binding site to the binding constant for the specific binding site for the TetR regulator; 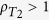 represents the capacity for regulation of the *lac* promoter by TetR repressor.

TetR repression of the λ promoter is represented mathematically as


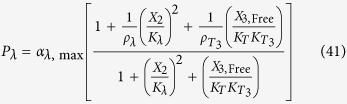


where 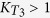 is the ratio of the binding constant for the non-target *λ* promoter binding site to the binding constant for the specific binding site for the TetR regulator; 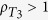 represents the capacity for regulation of the *λ* promoter by TetR repressor.

λ CI repression of the *lac* promoter is represented mathematically as


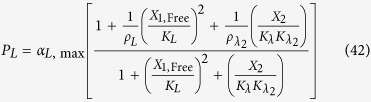


where 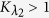 is the ratio of the binding constant for the non-target *lac* promoter binding site to the binding constant for the specific binding site for the λ CI regulator; 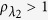 represents the capacity for regulation of the *lac* promoter by λ CI repressor.

λ CI repression of the *tet* promoter is represented mathematically as


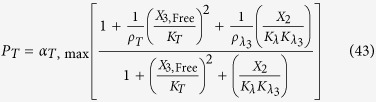


where 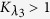
is the ratio of the binding constant for the non-target *tet* promoter binding site to the binding constant for the specific binding site for the λ CI regulator; 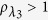 represents the capacity for regulation of the *tet* promoter by λ CI repressor.

#### Model-Discrimination Phase

Our strategy was to analyze the single mechanistically extended circuits to identify minimal hypotheses that could account for the experimental data. The TetR-primary and LacI-primary circuitry, extended with each of the single mechanistic extensions, were analyzed using the 4-step approach outlined for the conventional model of the TetR-primary and LacI-primary circuitry. The results of the model discrimination phase for each of the hypotheses, which take into consideration all basic landmarks involving stable and unstable features, are shown in Table 1.

#### Identifying individual sets of parameter values with results that mimic the experimental data for each circuit

Columns 2 and 3 of Table 1 show that 4 of 9 hypotheses for the TetR-primary circuit and 6 of 9 hypotheses for the LacI-primary circuit are capable of realizing the appropriate logic at the architectural level. The hypotheses that can generate the respective logic functions for the TetR-primary and the LacI-primary circuits are transcriptional read-through, non-specific repression, LacI repression of the λ promoter and TetR repression of the λ promoter. However, if we assume the experimental conditions were well controlled, it is reasonable to expect that there should be a single hypothesis that can explain all experimental observations with a single set of values for the parameters.

#### Identifying a unified set of parameter values with results that mimic the experimental data

We have previously shown how the System Design Space method can be used to automatically identify a common set of parameter values such that the qualitatively distinct phenotypes necessary to represent the global function of a model are simultaneously realized, a technique called ‘co-localization’ in the more general context^12^. Here, we extend this capability of the System Design Space method to deal not only with phenotypes of a single model, but with phenotypes across multiple models by determining whether a common set of parameter values exists such that the qualitatively distinct phenotypes for both the TetR-primary and LacI-primary circuits are all realized simultaneously. We apply this method to determine if a single set of parameter values exist such that both the TetR-primary and LacI-primary circuits, extended according to each of the 9 hypotheses in Table 1, can exhibit the NAND + NIF1 and NOR logic functions, respectively.

The results of this test, summarized in the last column of Table 1, show that there is no single hypothesis that is able to explain both sets of experimental data simultaneously. Therefore, independent of specific values for the parameters, we have shown that none of these hypotheses can account for the experimental data. Taken together, our 4-step approach has eliminated all 9 of these hypotheses, which are thus poor candidates for further experimental testing.

### Dual Mechanistic Extensions of the Conventional Models

Our analysis thus far has analyzed a total of 9 hypotheses involving individual mechanistic extensions for the TetR-primary and LacI-primary circuits and found that none of the hypotheses were able to reproduce the experimentally observed results. However, any of these individual mechanisms might still be contributing to the observed results by acting in concert with other mechanisms and producing emergent new phenotypes. Here, we consider hypotheses where the conventional model of the TetR-primary and LacI-primary circuits is extended by all combinations of 2 mechanisms from the 9 we analyzed previously. The result is a total of 30 additional hypotheses involving dual mechanistic extensions. As in the case of the single mechanistic extensions, we proceed with the 4-step analysis of the dual mechanistic extensions in the model-dependent phase.

#### Model-Discrimination Phase

In the model-discrimination phase of dual mechanistically extended circuits we examine the models at both the architectural and biological levels. A summary of the results is shown in Table 2.

#### Architectural level comparisons

The dual extended circuits were analyzed first at the architectural level to identify those that had the capacity to reproduce both sets of experimental data. Columns 2–4 in Table 2 show the results of Step 3 and Step 4 of the 4-step approach applied to all dual mechanistically extended circuits at the architectural level.

Columns 2 and 3 of Table 2 show that 21 of 30 dual hypotheses for the TetR-primary circuit, 29 of 30 dual hypotheses for the LacI-primary circuit, and 20 of 30 dual hypotheses for both circuits considered together are capable of producing the appropriate logic at the architectural level.

We apply the next step of our strategy to determine whether there is a unifying set of values for the parameters such that both circuits are able to realize the appropriate binary logic functions at the architectural level. Column 4 of Table 2 shows that there are only 5 of 30 dual hypotheses that can simultaneously realize both sets of experimental data at the architectural level.

#### Biological level comparisons

The analysis up to this point has screened through multiple hypotheses to identify those that have the potential to reproduce the observed experimental data at the architectural level. This level of discrimination is very powerful because it does not make any assumption about parameter values, as long as the architectural features are maintained. However, even if a hypothesis can reproduce the appropriate logic functions at the architectural level, there is no guarantee that it can do so with values for the parameters that are physically or biologically realizable or relevant.

Here, we start with the 5 hypotheses from the previous subsection that can realize the appropriate logic at the architectural level. The analysis was repeated to identify the hypotheses that could succeed at all 4 steps of the approach at the biological level by imposing the constraints in Table 3. The results of our model-discrimination phase for the dual hypotheses are as follows: the TetR-primary and the LacI-primary circuits, extended with all 5 of the architecturally realizable dual mechanisms, are individually capable of producing the appropriate logic at the biological level. However, when we applied our method to determine whether they can realize the appropriate logic simultaneously, we found this to be true for only 1 of the 5 dual hypotheses, as shown in the last column of Table 2. The hypothesis that we found capable of generating both sets of experimentally observed logic functions involves LacI and TetR repression of the λ promoter.

#### Identifying a unified set of parameter values that mimic the experimental data

The results of Step 4 of our approach included 364 unique combinations of phenotypes for the TetR-primary and LacI-primary circuits taken together and extended with both LacI and TetR repression of the λ promoter. Once we determined that a subset of phenotypes had the correct levels for both extended TetR-primary and LacI-primary circuits, we identified the phenotypes that were valid at the +/+, +/−, −/+ and −/− conditions. We then applied the method of phenotype co-localization for the 8 phenotypes at these conditions, 4 for the TetR-primary circuit and 4 for the LacI-primary circuit, and imposed constraints on the values of GFP for each of the phenotypes so they would be consistent with the quantitative data observed experimentally. In addition, we imposed constraints on values for the concentrations of IPTG and aTc to ensure that they would be localized at the correct values to be consistent with the experimental observations.

**Figure 8:**
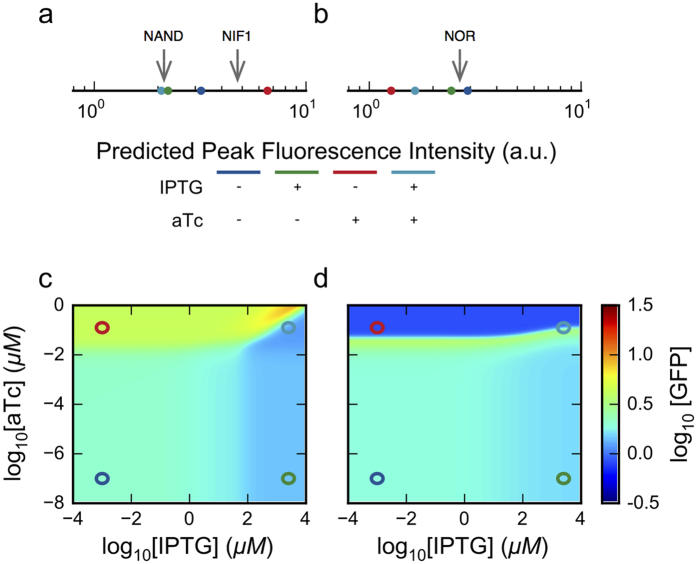
Predicted landscapes that realize the observed logic of the TetR-primary [(**a,c**)] and LacI-primary [(**b,d**)] circuits extended to include LacI and TetR repression of the λ promoter. (**a,b**) Peak GFP fluorescence intensity (population average), normalized with respect to lowest fluorescence intensity for both circuits, represented by the closed circle on the line at values consistent with the experimentally reported values of IPTG and aTc, as shown in the key below. Arrows above the line indicate putative threshold concentrations that realize particular binary logic functions. (**c,d**) Steady state concentration of GFP, represented by color on the z-axis, for (**c**) the TetR-primary circuit and (**d**) the LacI-primary circuit The predicted values for the parameters that yield these data are shown in Table 3.

One example showing the simultaneous realization of both binary logic functions, determined through our automated System Design Space methods^12^, is shown in Fig. 8. The GFP expression profile for the TetR-primary circuit can exhibit a NAND binary logic if the appropriate threshold for GFP concentration is chosen; however, the pattern is more similar to that of a NIF1 binary logic function and is thus consistent with what has been observed experimentally^7^. The GFP expression profile for the LacI-primary circuit also replicates the NOR logic gate observed experimentally. Although these profiles are not exact replicates of the observed GFP profiles, we have nonetheless shown a definitive example replicating the qualitative aspects of the synthetic constructs through an automated method that does not rely on parameter sampling or direct manipulation of any of the parameters.

## Discussion

Determining whether specific biochemical mechanisms are able to reproduce a set of qualitative experimental results typically involves understanding a complex non-linear model that is analytically intractable. One could proceed empirically by using a numerical sampling approach. However, if such an approach does not produce a concrete example of the observed results, then there would be no proof of the model’s capacity – or lack thereof – to realize the experimental observations. If we are to show that a model cannot exhibit a particular result, then we must do so in a manner that is independent of specific parameter values. Here, we have presented a strategy that can be used to discriminate against models that do not have the potential to realize the experimentally observed results.

This new strategy can discriminate among mechanistic models based on their capacity to reproduce experimental observations. A large volume of available data consists of discrete observations, where a small number of entities increase or decrease in relation to some reference. This usually implies that a large number of models can account for the experimental observations. Using traditional approaches to discriminate among the models involves finding a model that is the ‘best’ fit – hence discrimination becomes parameter dependent.

Model parameterization is no easy task and it involves searching the literature for parameter values and fitting experimental data. There are significant problems with each of these. A literature search for parameter values is often looking for a ‘needle in a haystack’, with the added complexity that the results are highly dependent on the experimental context and specific conditions. Parameter fitting is a challenge when the parameter space is large and the data are sparse and noisy, where a ‘best’ fit is ambiguous due to the many degrees of freedom.

The three-phase strategy we have presented has a distinguishing property: we are able to exclude possibilities in a manner that is independent of parameter values, even when data are sparse and parameter values unknown. In the model-independent phase, the task is to obtain a description of discrete experimental observations that is based on necessary basic landmarks, and is independent of any model. We have demonstrated that there are a finite number of these necessary basic landmarks for binary logic functions. In the model-dependent phase, the task is to identify the qualitatively distinct phenotypes of a model, independent of specific parameter values and independent of the experimental data. The collection of qualitatively distinct phenotypes characterizes the model’s phenotypic repertoire. We have previously demonstrated that there are a finite number of these phenotypes as well. In the model-discrimination phase, results from the two previous phases are combined to identify hypotheses that can be further tested.

The key assumptions of this approach are that the discrete experimental data are in regimes where there is only one stable state of the system and that the underlying landscapes are continuous functions of the input variables with at least one non-zero stable state throughout. The latter of these assumptions should be valid for most gene regulatory circuits and the former can be determined experimentally by looking at populations of individual cells, as was done in the experimental study by using FACS measurements^7^.

As an application of this strategy we analyzed a total of 80 mathematical models involving two synthetic gene circuits and determined the models’ ability to reproduce experimentally observed data. We first demonstrated that the conventional models – consisting of mechanisms as they operate in their native context – were not able to generate the experimentally observed results, regardless of parameter values. Thus, other mechanisms that are negligible in the native context must be contributing significantly in the synthetic context. We first considered 9 such mechanistic extensions of the conventional model that are well established, and 30 dual combinations thereof: transcriptional read-through, specific binding of LacI-IPTG to operator DNA, non-specific binding of LacI to backbone DNA, and regulator specific cross-talk involving non-target repression by LacI, TetR and λ CI for each of the possible promoters.

The experimental data used here consist only of values at the four extreme values of the inputs, and the logic functions exhibited are qualitative. The test of the models is to determine whether or not they are capable of generating the appropriate logic functions. Our analysis has shown that among the 40 different hypotheses for each circuit, only 5 have the potential to reproduce the observed logic functions for both circuits with some combination of values for the parameters. Furthermore, our methods provide semi-quantitative results that can be compared with the experimental data (Fig. 8). By imposing bounds considered to be biologically relevant on the values for the parameters, we find that only 1 hypothesis among the 40 is consistent with the observed quantitative experimental data. The only hypothesis among those tested that can reproduce the qualitative nature of the experiments within what we have deemed to be biologically relevant values involves a combination of LacI repression of the λ promoter and TetR repression of the λ promoter. There are additional strategies to further discriminate among the surviving models and to quantitatively characterize those that are most successful.

First, our methods involve all the parameters and variables of the full models being tested, so these methods can be used in combination with any other method to explore the common parameter space. For any model that successfully passes the qualitative tests, our methods predict representative quantitative values for the underlying parameters of the model. If desired, these parameter values can be further refined by any number of parameter estimation routines when applied to the full model and using the predicted values as starting points. However, since there are only four data points, there are likely to be several estimates capable of “fitting” the data.

Second, and more productive, would be to generate additional experimental data consisting of titrations of increasing and decreasing values of the inputs, as in Williams *et al.*^37^. This would provide additional landmarks and local couplings that could be used to further discriminate qualitatively among the models. For example, one might expose graded or discontinuous behaviors with particular thresholds (see Figures S1 and S2) at intermediate values of the inputs that could only be generated by particular models. Moreover, the additional qualitative tests would provide more rigorous predictions of representative quantitative values for the underlying parameters of the models. Again these starting values can be further refined by a variety of additional methods, and the additional experimental data also would provide more power for any attempt to statistically estimate a best-fit set of parameter values.

By analyzing the conventional models for the synthetic gene circuits and by considering additional hypotheses, we have gained insight into key mechanisms that are likely to be influencing the behavior of these constructs. In so doing we have provided an example of a rigorous mathematical analysis that has provided an understanding of the relationship between a conceptual design for a synthetic construct and its physical realization. Our analysis has shown that even small effects can have large implications. For example, although the maximal rate of protein synthesis for the P^L^ promoter in Table 3 is much lower than that for the P^T^ promoter, it cannot be discounted as irrelevant. In fact, our analysis indicates that the interaction is absolutely necessary for the circuits to be able to reproduce the qualitative aspects of the observed behaviors. Furthermore, perhaps a more valuable result is the fact we have identified 35 of 40 hypotheses that are unlikely to reproduce the experimental data regardless of the values for the parameters.

The focus of our illustration has been to identify the simplest hypotheses that had the potential to reproduce the binary logic functions observed experimentally. Nonetheless, we have applied this approach to more complex hypotheses involving combinations of 3 and even 4 of the mechanisms that we have discussed here. Among these hypotheses we found other examples that could generate the appropriate binary logic function and one in particular, which involves the combination of transcriptional read-through plus TetR repression of the *lac* and *λ* promoters, very closely matches the quantitative aspects of the experimental data.

We also applied this approach to a hypothesis proposed by Kim and Tidor^38^, where the high concentrations of the ssra-tagged proteins in these circuits may overburden the cellular protease machinery. This adds an additional layer of regulation where proteins mutually inhibit their degradation through saturation of these enzymes. Our approach found that this hypothesis satisfied the necessary conditions simultaneously for both circuits at the architectural level, but could not do so at the biological level.

With this application we have demonstrated a novel approach that can be used to rapidly screen through multiple hypotheses without the need for previous knowledge of the specific values for the parameters; thereby focusing experimental effort on hypotheses that are feasible and away from those that our approach has shown are unlikely. The results of our analysis suggest critical experiments to test the feasible hypotheses. Ultimately, this approach can be applied to extract functional implications of hypothesized mechanisms in natural gene circuitry, as well as synthetic systems, that carry out binary logic functions other than those considered here.

## Additional Information

**How to cite this article**: Lomnitz, J. G. and Savageau, M. A. Rapid Discrimination Among Putative Mechanistic Models of Biochemical Systems. *Sci. Rep.* **6**, 32375; doi: 10.1038/srep32375 (2016).

## Supplementary Material

Supplementary Information

## Figures and Tables

**Table 1:** Model discrimination of mechanistically extended models at the architectural level.

Extended Mechanism	Individually Realizable		Simultaneously Realizable
	TetR-primary	LacI-primary	
Read-through	Y	Y	N
LacI-IPTG specific repression	Y	Y	N
LacI Non-specific repression	N	Y	—
LacI repression of TetR target	N	N	—
LacI repression of λ target	Y	Y	N
TetR repression of LacI target	N	N	—
TetR repression of λ target	Y	Y	N
λ repression of LacI target	N	Y	—
λ repression of TetR target	N	N	—

Individual realization is either yes (Y) or no (N), and simultaneous realization is yes (Y), no (N), or not determined (—) when one or both of the individual circuits cannot be realized.

**Table 2:** Model discrimination and simultaneous realization of circuits extended by two mechanisms at both the architectural and biological level.

Extended Mechanism	Architectural			Biological
	Individually Realizable		Simultaneously Realizable	
	TetR-primary	LacI-primary		Simultaneously Realizable
Read-through + LacI-IPTG specific repression	Y	Y	N	—
Read-through + LacI non-specific repression	Y	Y	N	—
Read-through + LacI repression of TetR target	Y	Y	N	—
Read-through + LacI repression of λ target	Y	Y	Y	N
Read-through + TetR repression of LacI target	Y	Y	N	—
Read-through + TetR repression of λ target	Y	Y	N	—
Read-through + λ repression of LacI target	Y	Y	N	—
Read-through + λ repression of TetR target	Y	Y	N	—
LacI-IPTG specific repression + LacI non-specific repression	Y	Y	N	—
LacI-IPTG specific repression + LacI repression of TetR target	N	Y	—	—
LacI-IPTG specific repression + LacI repression of λ target	Y	Y	N	—
LacI-IPTG specific repression + TetR repression of LacI target	N	Y	—	—
LacI-IPTG specific repression + TetR repression of λ target	Y	Y	Y	N
LacI-IPTG specific repression + λ repression of LacI target	N	Y	—	—
LacI-IPTG specific repression + λ repression of TetR target	N	Y	—	—
LacI repression of TetR and λ targets	Y	Y	N	—
LacI (TetR) repression of TetR (LacI) target	N	Y	—	—
LacI (TetR) repression of TetR (λ) target	Y	Y	Y	N
LacI (λ) repression of TetR (LacI) target	N	Y	—	—
LacI (λ) repression of TetR (TetR) target	N	Y	—	—
LacI (TetR) repression of λ (LacI) target	Y	Y	N	—
LacI (TetR) repression of λ (λ) target	Y	Y	Y	Y
LacI (λ) repression of λ (LacI) target	Y	Y	N	—
LacI (λ) repression of λ (TetR) target	Y	Y	N	—
TetR repression of LacI and λ target	Y	Y	N	—
TetR (λ) repression of LacI (LacI) target	N	Y	—	—
TetR (λ) repression of LacI (TetR) target	Y	N	—	—
TetR (λ) repression of λ (LacI) target	Y	Y	N	—
TetR (λ) repression of λ (TetR) target	Y	Y	Y	N
λ repression of LacI and TetR target	N	Y	—	—

Individual realization is either yes (Y) or no (N), and simultaneous realization at either the architectural or biological level is yes (Y), no (N), or not determined (—) when one or both of the individual circuits cannot be realized.

**Table 3:** Comparison between estimated reference values for the parameters and automatically determined values for simultaneous realization of phenotypes.

Parameter	Experimental	Computed	Biological Range	Units
*α*_*L*,max_	1 (see^39^)	2.27 × 10^−3^	0.001–10000	μM min^−1^
*α*_*T*,max_	0.6 (see^40^)	0.58	0.0001–1000	μM min^−1^
*α*_*λ*,max_	1 (see^39^)	1.00	0.0001–1000	μM min^−1^
*ρ*_*L*_	620 (see^32^)	108.18	>1	*
*ρ*_*L*2_	—	N/A	>1	*
*ρ*_*L*3_	—	11.18	>1	*
*ρ*_*T*_	5300 (see^32^)	5773.5	5000–7000	*
*ρ*_*T2*_	—	N/A	>1	*
*ρ*_*T3*_	—	30	>1	*
*ρ*_*λ*_	70	57.73	>50	*
*ρ*_*λ*2_	—	N/A	>1	*
*ρ*_*λ*3_	—	N/A	>1	*
*Ρ*_*ns*_	—	N/A	>1	*
*Ρ*_*LI*_	—	N/A	>1	*
*K*_*L*_	3.5 × 10^−7^ (see^34^)	8.66 × 10^−5^	1 × 10^−10^–1 × 10^−4^	μM
*K*_*L*2_	—	N/A	>1	μM
*K*_*L*3_	—	1.15	>1	μM
*K*_*T*_	1.79 × 10^−4^ (see^41^)	2.59 × 10^−4^	1 × 10^−6^–1 × 10^−1^	μM
*K*_*T2*_	—	N/A	>1	*
*K*_*T3*_	—	2.04	>1	*
*K*_*λ*_	8 × 10^−4^ (see^39^)	8.66 × 10^−3^	1 × 10^−6^–1 × 10^−2^	μM
*K*_*λ*2_	—	N/A	>1	μM
*K*_*λ*3_	—	N/A	>1	μM
*K*_*ns*_	1 × 10^5^–1 × 10^10^ (see^35^)	N/A	1 × 10^5–^1 × 10^10^	μM
*K*_*LI*_	1000 (see^34^)	N/A	>1 × 10^3^	μM
*k*_*I*_	1.2–1.3 (see^3^,^34^)	1.15	0.01–100	μM
*k*_*a*_	1 × 10^−5^ (see^42^)	0.0086	1 × 10^−6^–1 × 10^−1^	μM
*β*_*1*_	0.02310 (see^7^)	0.0217	0.002–0.025	min^−1^
*β*_*2*_	0.02310 (see^7^)	0.0217	0.002–0.025	min^−1^
*β*_*3*_	0.02310 (see^7^)	0.0023	0.002–0.025	min^−1^
*β*_*4*_	0.02310 (see^7^)	0.0217	0.002–0.025	min^−1^
*σ*_1_	0.01	N/A	<0.01	*
*σ*_2_	0.01	N/A	<0.01	*
*σ*_3_	0.01	N/A	<0.01	*

Parameter values taken from the literature were either estimated, experimentally measured or absent (—). Parameter values computed in this study were determined automatically for the single hypothesis that satisfied both experimental observations simultaneously. Parameters not participating in the model are marked as Not Applicable (N/A). Biological Range indicates ranges for the parameters considered to be biologically relevant. The units for the parameters are shown using the appropriate symbols, or marked as dimensionless (*).
