# Supplementary material for: Rapid Discrimination Among Putative Mechanistic Models of Biochemical Systems
Source: Sci Rep. 2016 Aug 31;6:32375. doi: 10.1038/srep32375 (PMC5006174; doi:10.1038/srep32375)
Supplement: Supplementary Information [file srep32375-s1.pdf]

SUPPLEMENTARY FIGURES

Rapid Discrimination Among Putative Mechanistic Models of Biochemical Systems

Jason G. Lomnitz[1] and Michael A. Savageau[1,2,*].

[1]Department of Biomedical Engineering, and [2]Department of Microbiology & Molecular Genetics, University of California, Davis, CA 95616 USA

* To whom correspondence should be addressed. Tel: +01 530 754 7350; Fax: +01 530 754 5739; Email: masavageau@ucdavis.edu

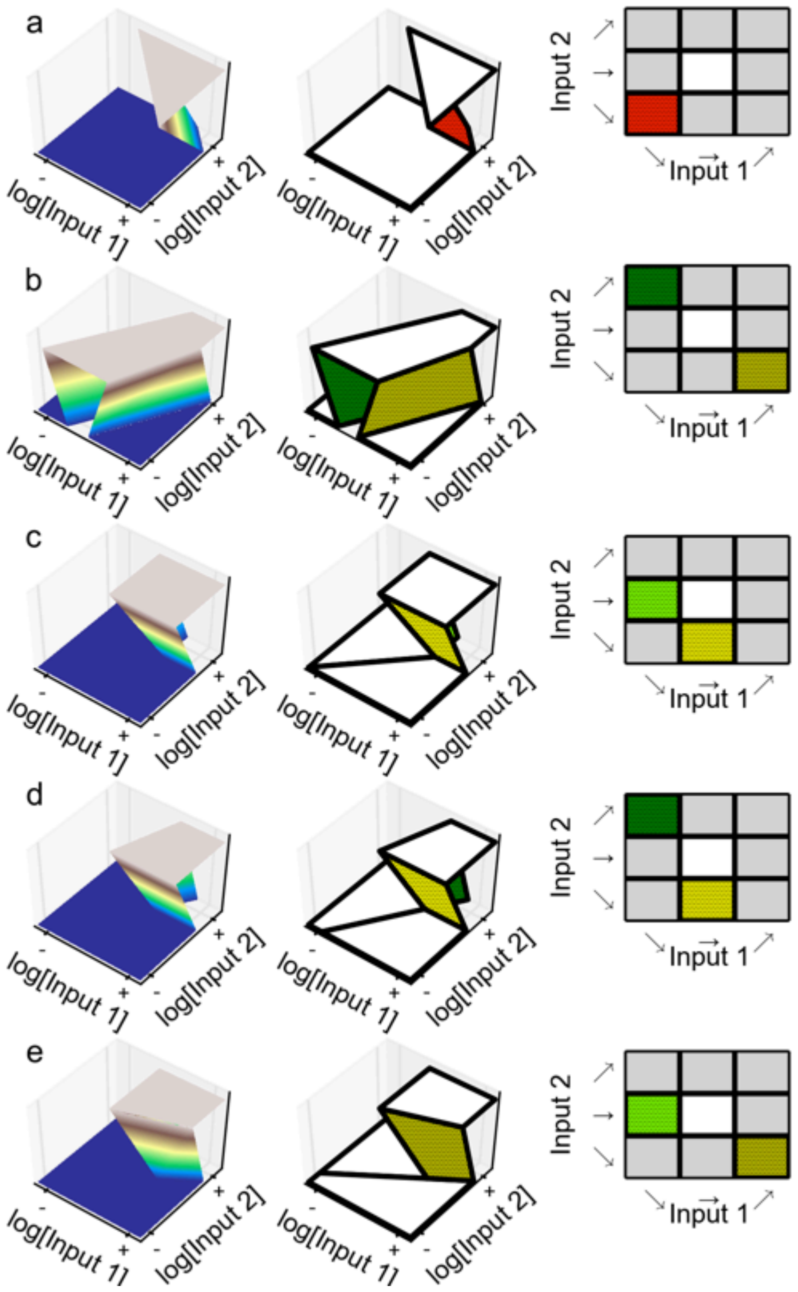

Figure S1. Basic landmarks, features and local couplings for the AND logic function involving unstable transitions. Left panels: Basic landmarks with the output value on the z-axis. The '–' and '+' symbols on the axes correspond to the low and high input values of the discrete experimental data, respectively. Center panels: Features on the z-axis corresponding to the panels on the left. Right panels: Local couplings between input and output are inversely coupled ↘, uncoupled →, and directly coupled ↗. Colored squares indicate the local couplings in the basic landmark as shown by the key in Figure 3;

white squares indicate local couplings in the basic landmark where the output is uncoupled from both inputs; gray squares represent local couplings that are not present in the basic landmark.

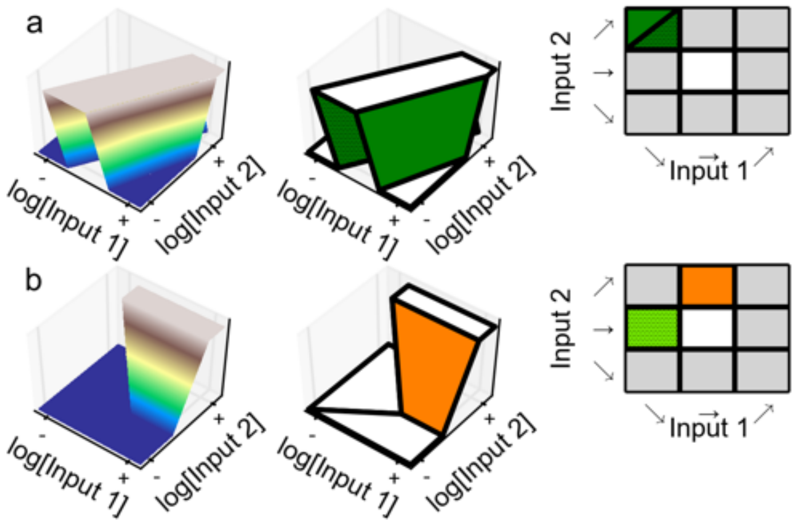

Figure S2. Basic landmarks, features and local couplings for the AND logic function involving one stable and one unstable transition. See captions of Figures 3 and 4 for details.

Appendix:

Proof of Five Basic Landmarks involving stable features for an AND Logic Function

Definition. Let b_i be a basic landmark that can generate an AND logic function.

Definition. Let f_i be the set of features that comprise b_i such that f_{ij} is the j-th feature of b_i. The local coupling of $f_{ij}, \forall j = 1,\ldots,n$ is defined by $\partial \log z_{ij} / \partial \log x$ and $\partial \log z_{ij} / \partial \log y$, where z is a single-valued function corresponding to the output concentration, such that $z := g(x,y)$ where x is the first input signal and y is the second input signal. The fixed point of $f_{ij}, \forall j = 1,\ldots,n$ is assumed to be stable.

Theorem. Let b_1, b_2, b_3, b_4, b_5 be basic landmarks that can generate an AND logic function in R^2, such that

$$b_1 \left\{ \quad \partial \log z_{11} / \partial \log x > 0 \quad \partial \log z_{11} / \partial \log y > 0 \right.$$

$$b_2 \begin{cases} \partial \log z_{21} / \partial \log x > 0 & \partial \log z_{21} / \partial \log y = 0 \\ \partial \log z_{22} / \partial \log x = 0 & \partial \log z_{21} / \partial \log y > 0 \end{cases}$$

$$b_3 \begin{cases} \partial \log z_{21} / \partial \log x > 0 & \partial \log z_{21} / \partial \log y = 0 \\ \partial \log z_{22} / \partial \log x < 0 & \partial \log z_{21} / \partial \log y > 0 \end{cases}$$

$$b_4 \begin{cases} \partial \log z_{21} / \partial \log x > 0 & \partial \log z_{21} / \partial \log y < 0 \\ \partial \log z_{22} / \partial \log x = 0 & \partial \log z_{21} / \partial \log y > 0 \end{cases}$$

$$b_5 \begin{cases} \partial \log z_{21} / \partial \log x > 0 & \partial \log z_{21} / \partial \log y < 0 \\ \partial \log z_{22} / \partial \log x < 0 & \partial \log z_{21} / \partial \log y > 0 \end{cases}$$

Then, $B = \{b_1, b_2, b_3, b_4, b_5\}$ is the complete set of basic landmarks that can generate an AND logic function for a continuous system with only stable steady states.

Proof.

Suppose b_6 is a basic landmark. Let $f_6 = \{f_{61},\ldots, f_{6n}\}$ be the set of n individual features that comprise b_6. We must show that $b_6 \in B$.

For an AND logic function, there must be an output value, $z(x_0, y_0 + \Delta y)$, such that

$$z(x_0, y_0 + \Delta y) < z(x_0 + \Delta x, y_0 + \Delta x) \tag{A.1}$$

because the function is continuous and all features are stable, there is a positive gradient in x such that $\partial \log z / \partial \log x > 0$ at some x_C such that $x_0 < x_C < x_0 + \Delta x$. Similarly, there is a $z(x_0 + \Delta y, y_0)$ such that

$$z(x_0 + \Delta x, y_0) < z(x_0 + \Delta x, y_0 + \Delta x) \tag{A.2}$$

again, there is a positive gradient in y such that $\partial \log z / \partial \log y > 0$ at some y_C such that $y_0 < y_C < y_0 + \Delta y$.

For b_6 to be a basic landmark, there must be a feature, f_{ij}, such that

$$\partial \log z_{ij} / \partial \log x > 0 \quad \text{(A.3)}$$

and a feature, f_{ik}, such that

$$\partial \log z_{ik} / \partial \log y > 0 \quad \text{(A.4)}$$

If there is a single feature that satisfies conditions (A.3) and (A.4), such that $j = k$ and thus $f_{ij} \equiv f_{ik}$, then f_{ij} has the following local couplings,

$$\partial \log z_{ij} / \partial \log x > 0 \quad \partial \log z_{ij} / \partial \log y > 0 \quad \text{(A.5)}$$

These local couplings are the same as the local couplings for b_1. If $n > 1$, then the landmark is non-basic and features can be removed until only f_{ij} remains. Therefore, for b_6 to be basic and $f_{ij} \equiv f_{ik}$ to be true, then $n = 1$ and $b_6 \equiv b_1$.

However, if $j \neq k$, then there must be a feature, f_{ij}, such that

$$\partial \log z_{ij} / \partial \log x > 0 \quad \partial \log z_{ij} / \partial \log y \leq 0 \quad \text{(A.6)}$$

and a feature, f_{ik}, such that

$$\partial \log z_{ik} / \partial \log y > 0 \quad \partial \log z_{ik} / \partial \log y \leq 0 \quad \text{(A.7)}$$

If $n > 2$, then the landmark is non-basic and features can be removed until only the pair f_{ij} and f_{ik} remains. Therefore, for b_6 to be basic and $j \neq k$ to be true, then $n = 2$.

If $\partial \log z_{ij} / \partial \log y = 0$ in condition (A.6) and $\partial \log z_{ik} / \partial \log x = 0$ in condition (A.7), then these local couplings are the same as the local couplings for b_2 and so $b_6 \equiv b_2$.

If $\partial \log z_{ij} / \partial \log y = 0$ in condition (A.6) and $\partial \log z_{ik} / \partial \log x < 0$ in condition (A.7), then these local couplings are the same as the local couplings for b_3 and so $b_6 \equiv b_3$.

If $\partial \log z_{ij} / \partial \log y < 0$ in condition (A.6) and $\partial \log z_{ik} / \partial \log x = 0$ in condition (A.7), then these local couplings are the same as the local couplings for b_4 and so $b_6 \equiv b_4$.

The last possible combination of local couplings that satisfies conditions (A.6) and (A.7) is if $\partial \log z_{ij} / \partial \log y < 0$ and $\partial \log z_{ik} / \partial \log x < 0$. These local couplings are the same as the local couplings for b_5 and so $b_6 \equiv b_5$.

Therefore, for b_6 to be basic, then $b_6 \in \{b_1, b_2, b_3, b_4, b_5\}$ and so B is the complete set of basic landmarks for an AND logic function. The basic landmarks for a NAND, NOR and NIF1 logic are easily related to the landmarks for the AND binary function by simply reversing the sense of the inequalities for the logarithmic gain functions [for NIF1, only the inequality in condition (A.3)].
